# Supplementary material for: Glutamic acid–valine–citrulline linkers ensure stability and efficacy of antibody–drug conjugates in mice
Source: Nat Commun. 2018 Jun 28;9:2512. doi: 10.1038/s41467-018-04982-3 (PMC6023893; doi:10.1038/s41467-018-04982-3)
Supplement: Supplementary file 1 — Supplementary Information [file 41467_2018_4982_MOESM1_ESM.pdf]

Supplementary Information

Glutamic acid–valine–citrulline linkers ensure stability and efficacy of antibody–drug conjugates in mice

Anami et al.

Supplementary Figures

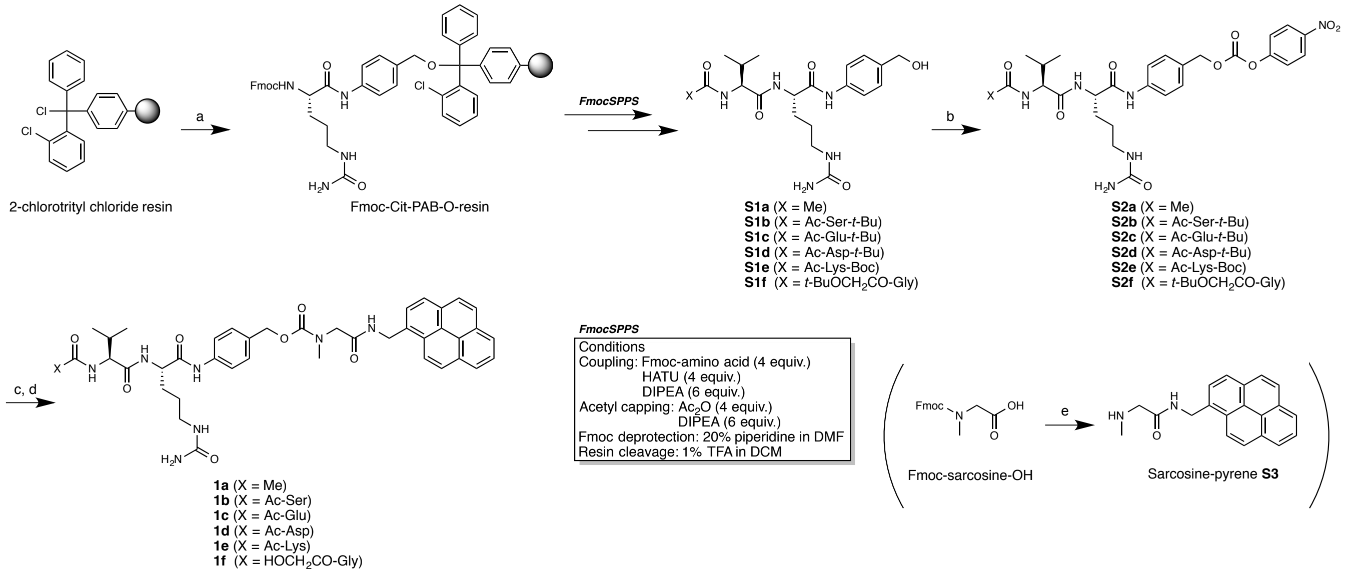

Supplementary Figure 1 Synthesis of pyrene probes **1a**–**f**. Reagents and conditions: (**a**) Fmoc-citrulline-PABOH, pyridine, 55 °C, overnight; (**b**) bis(4-nitrophenyl) carbonate, DIPEA (for **S2a**–**c**, and **S2e**) or DMAP (for **S2d** and **S2f**), DMF, room temp, overnight; (**c**) sarcosine-pyrene **S3**, DIPEA, DMAP, DMF, 37 °C, 2–4 h; (**d**) TFA, DCM, TIPS, room temp, 1 h for **1b**–**f**; (**e**) HATU, DIPEA, DMF, room temp, 2 h then 50% diethylamine/DMF, room temp, 1 h.

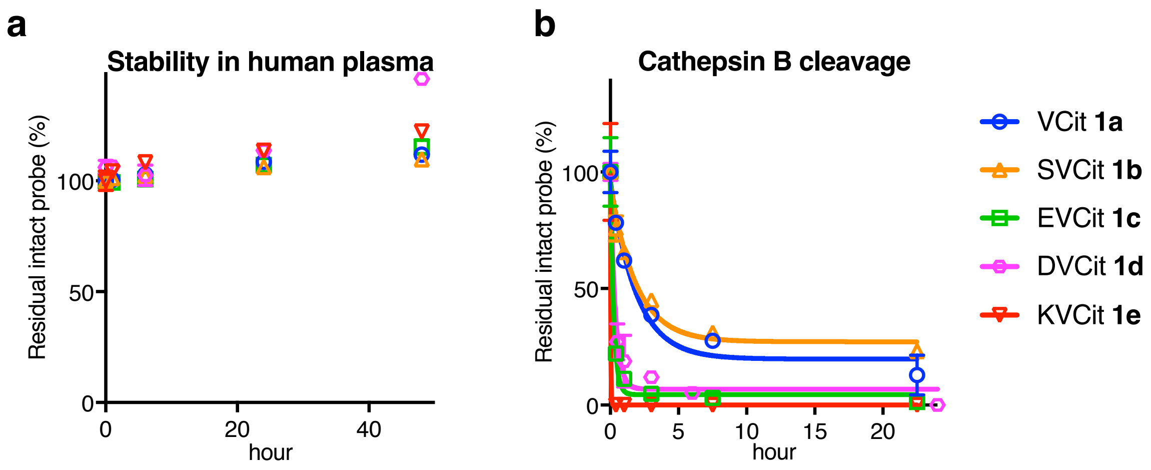

Supplementary Figure 2 Stability in human plasma and reactivity to cathepsin B-mediated cleavage. We tested probes containing VCit (**1a**, blue circle), SVCit (**1b**, orange triangle), EVCit (**1c**, green square), DVCit (**1d**, magenta hexagon), and KVCit (**1e**, red inversed triangle). (**a**) Stability of pyrene probes **1a**–**e** in human plasma at 37 °C. (**b**) Human cathepsin B-mediated cleavage of probes **1a**–**e** at 37 °C. Cleavage of each probe was monitored by LC/ESI-MS. Data shown are representative of more than two independent trials performed in technical duplicate. Error bars represent s.e.m. Curve fitting was performed using GraphPad Prism 7 software.

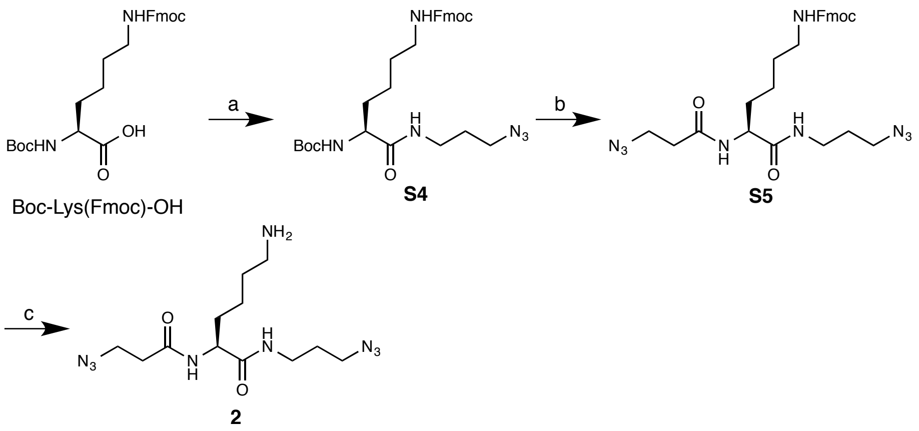

Supplementary Figure 3 Synthesis of branched linker **2**. Reagents and conditions: (**a**) 3-azidopropylamine, EDC-HCl, NHS, DMF, room temp, overnight; (**b**) 50% TFA/DCM, room temp, 1 h then 3-azidopropionic acid, EDC-HCl, NHS, DIPEA, DMF, room temp, overnight; (**c**) 50% diethylamine/DMF, room temp, 1 h.

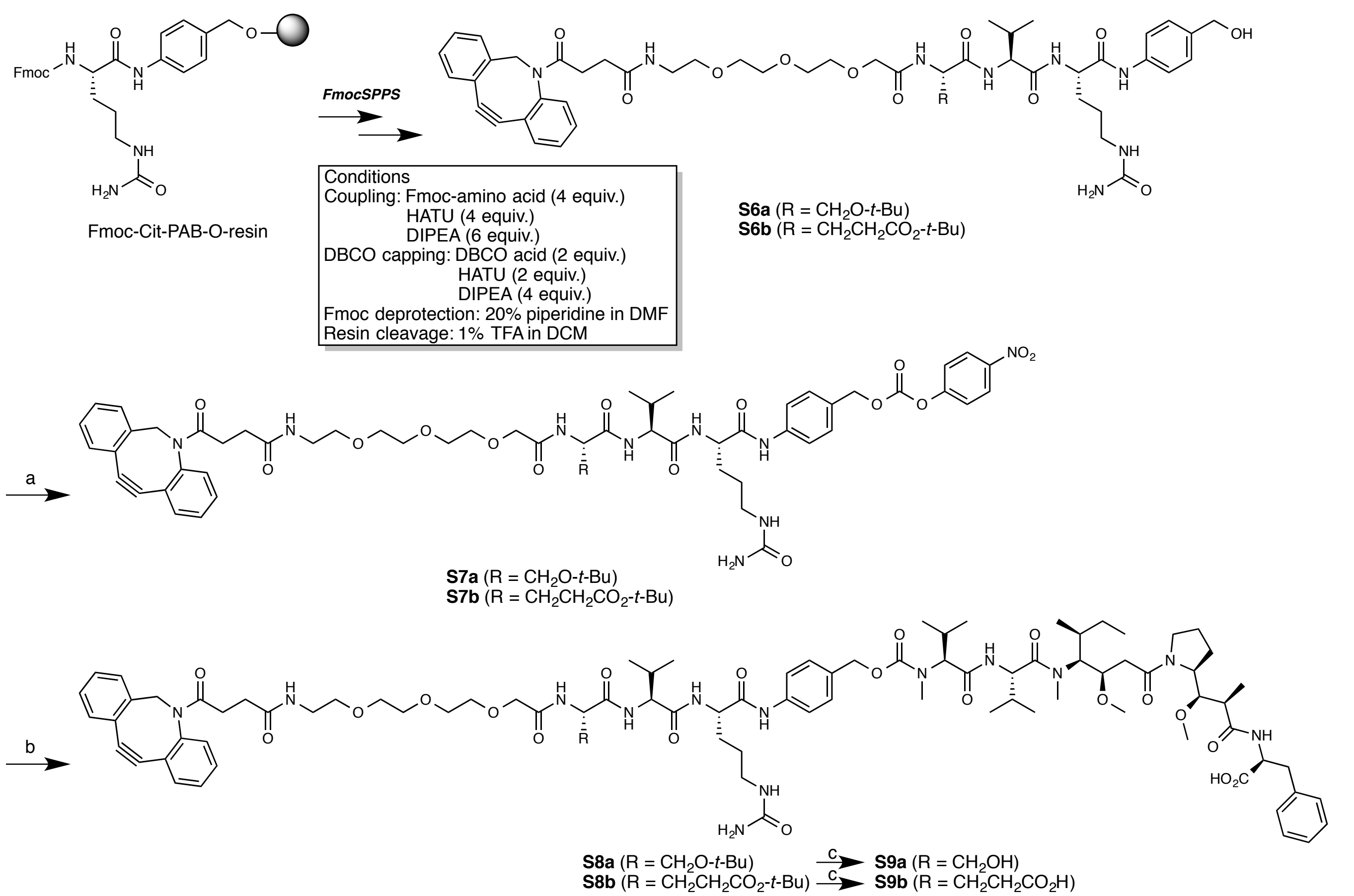

Supplementary Figure 4 Synthesis of DBCO–MMAF fragments **S9a** and **S9b**. Reagents and conditions: (**a**) bis(4-nitrophenyl) carbonate, DMAP, DMF, room temp, 2 h; (**b**) MMAF, DIPEA, HOAt, DMF, 37 °C, overnight; (**c**) 20% TFA/DCM, 0 ℃, 4 h.

a

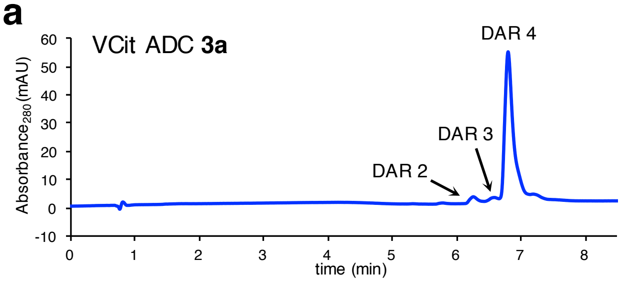

DAR	Time (min)	Area%
2	6.3	2.9
3	6.6	2.1
4	6.8	95.0

b

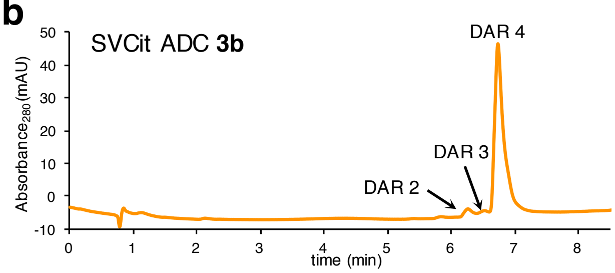

DAR	Time (min)	Area%
2	6.3	3.6
3	6.5	2.5
4	6.7	93.9

c

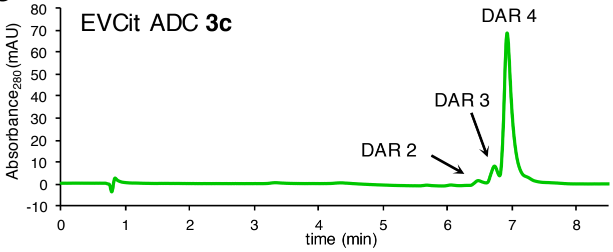

DAR	Time (min)	Area%
2	6.5	2.7
3	6.7	8.7
4	6.9	88.6

Supplementary Figure 5 Reverse-phase HPLC traces before SEC purification (UV: 280 nm). (**a**) VCit ADC **3a**, (**b**) SVCit ADC **3b**, and (**c**) EVCit ADC **3c**. The average DAR was determined to be 3.9 in all cases based on the peak areas of each DAR species.

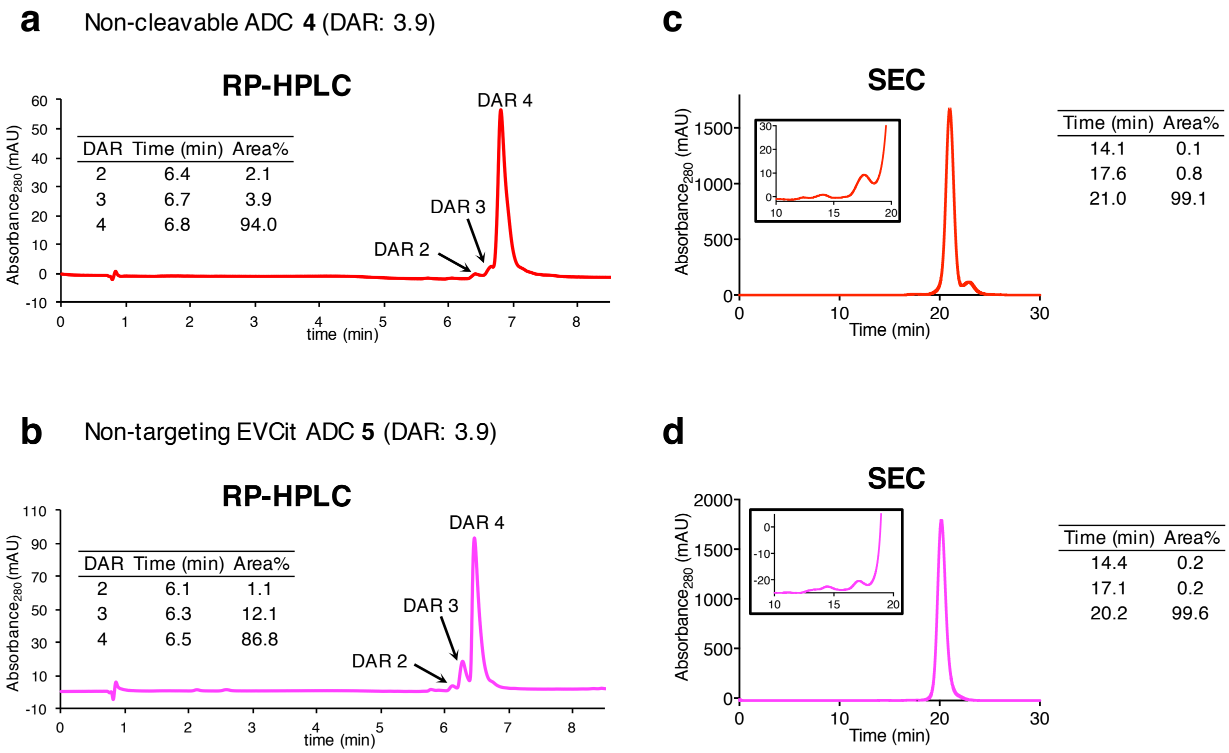

Supplementary Figure 6 Characterization of non-cleavable ADC **4** and non-targeting EVCit ADC **5**. Reverse-phase HPLC traces (UV: 280 nm) of (**a**) non-cleavable ADC **4** and (**b**) non-targeting EVCit ADC **5** before SEC purification. The average DAR was determined to be 3.9 in both cases based on the peak areas of each DAR species. SEC traces (UV: 280 nm) of (**c**) non-cleavable ADC **4** and (**d**) non-targeting EVCit ADC **5**. The monomer content of each ADC was >99%.

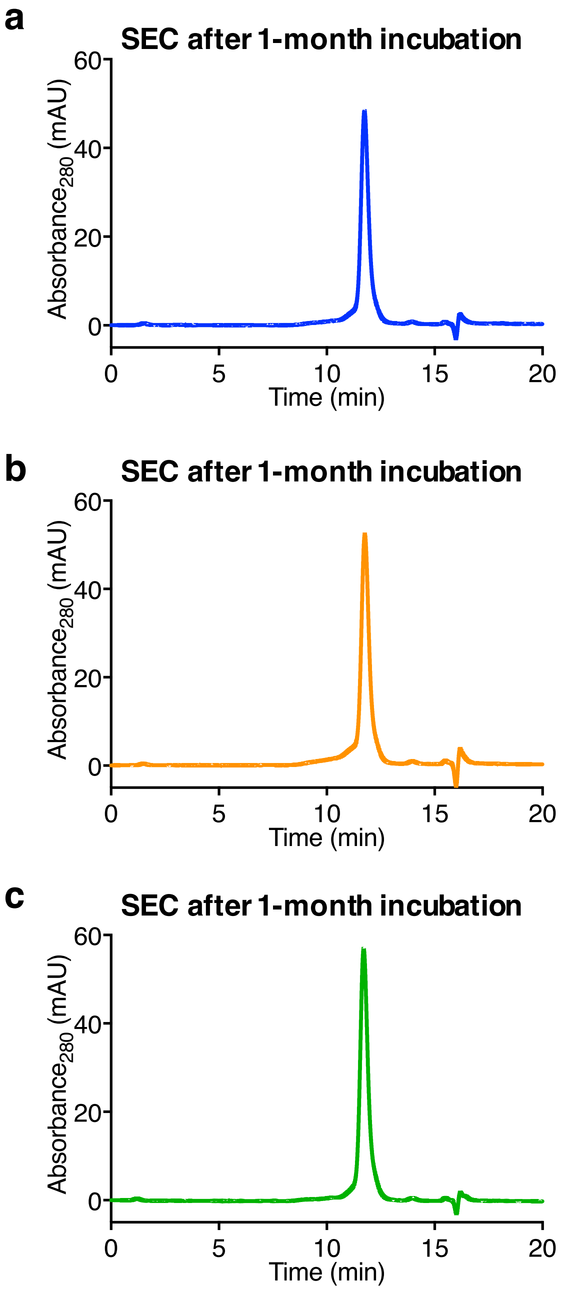

Supplementary Figure 7 SEC analysis of ADCs after incubation in PBS (pH 7.4) at 37 ºC for 1 month. (**a**) VCit ADC **3a**, (**b**) SVCit ADC **3b**, and (**c**) EVCit ADC **3c**. No significant aggregation was observed in all cases.

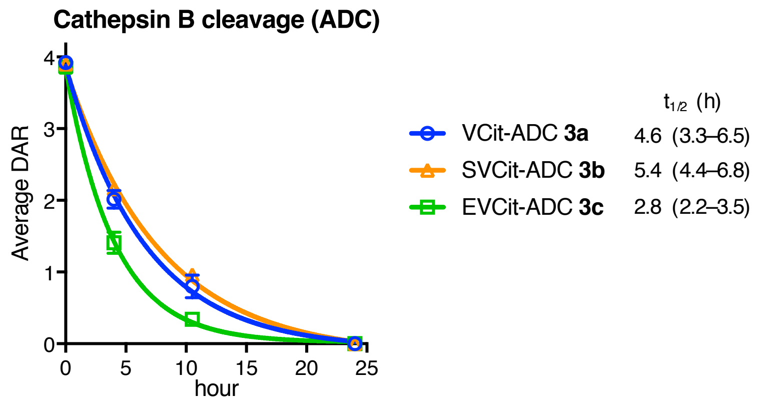

Supplementary Figure 8 Human cathepsin B-mediated cleavage of ADCs **3a**–**c** at 37 °C. The degree of loss of payload in each ADC was determined by HPLC. All assays were performed in more than twice in technical duplicate. Error bars represent s.e.m. (n = 2) and values in parentheses are 95% confidential intervals.

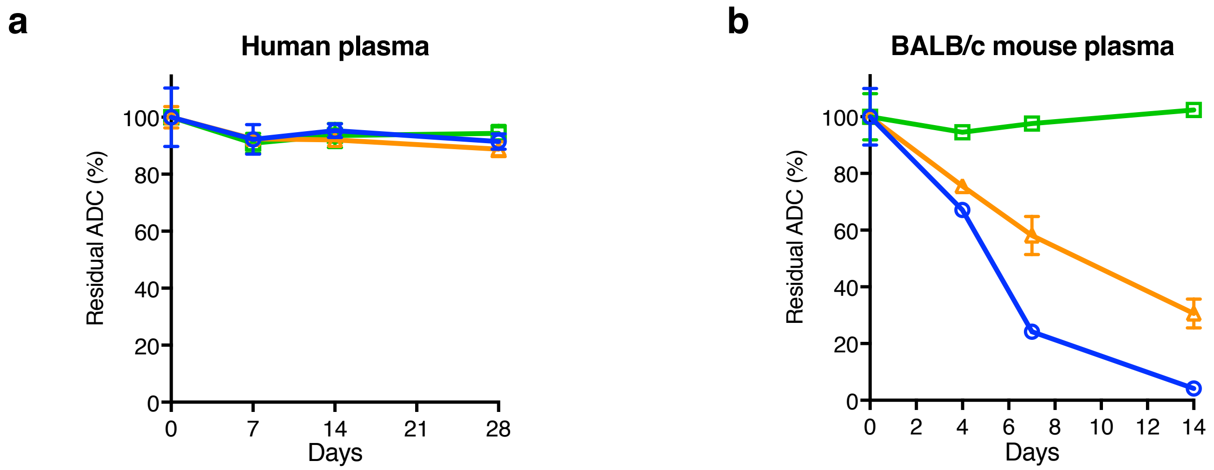

	Residual ADC (%)			
	0 day	7 day	14 day	28 day
VCit-ADC **3a**	100	92.3	95.3	91.5
SVCit-ADC **3b**	100	92.4	92.1	88.8
EVCit-ADC **3c**	100	90.9	93.6	94.4

	Residual ADC (%)			
	0 day	4 day	7 day	14 day
VCit-ADC **3a**	100	67.1	24.2	4.2
SVCit-ADC **3b**	100	75.6	58.1	30.6
EVCit-ADC **3c**	100	94.5	97.6	102.4

Supplementary Figure 9 Plasma stability of ADCs **3a**–**c**. The ADCs were incubated in (**a**) human plasma or (**b**) undiluted BALB/c mouse plasma at 37 °C (for ease of comparison, graphs previously shown in the manuscript are duplicated here). Concentrations of each ADC (conjugated only) were determined by sandwich ELISA. All assays were performed in triplicate and error bars represent s.e.m. Dorywalska et al. reported that a VCit-based tripeptide linker with a hydroxy-functionalized side chain at the P3 position (adjacent to V) showed the highest stability in mouse plasma among a panel of tripeptide linkers[1]. An ADC constructed using their linker retained about 84% payload conjugated at a highly solvent-accessible site after 4.5-day incubation in mouse plasma (estimated based on the reported figure). Given that hydroxy-functionalized SVCit ADC **3b** showed slightly lower but similar stability (75.6% payload retention after 4-day incubation), the SVCit ADC served in this study as a surrogate of the previously reported ADC with a similar stability profile.

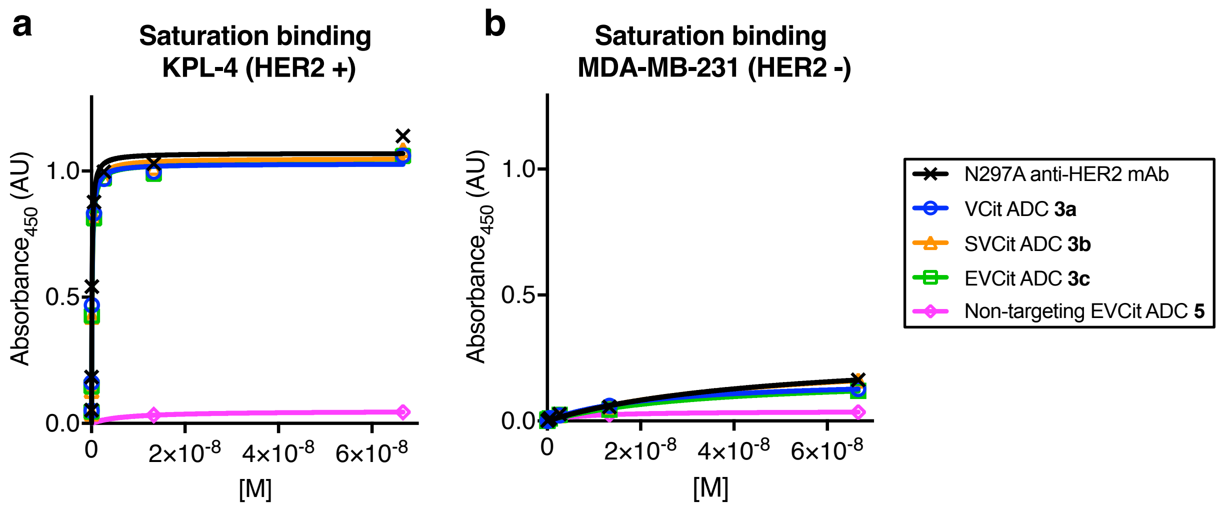

Supplementary Figure 10 Saturation-binding curves obtained by cell-based ELISA. All assays were performed in triplicate and error bars represent s.e.m. The unmodified N297A anti-HER2 antibody and ADCs **3a**–**c** bound to (**a**) KPL-4 cells (HER2 positive) with comparable binding affinities but not to (**b**) MDA-MB-231 cells (HER2 negative).

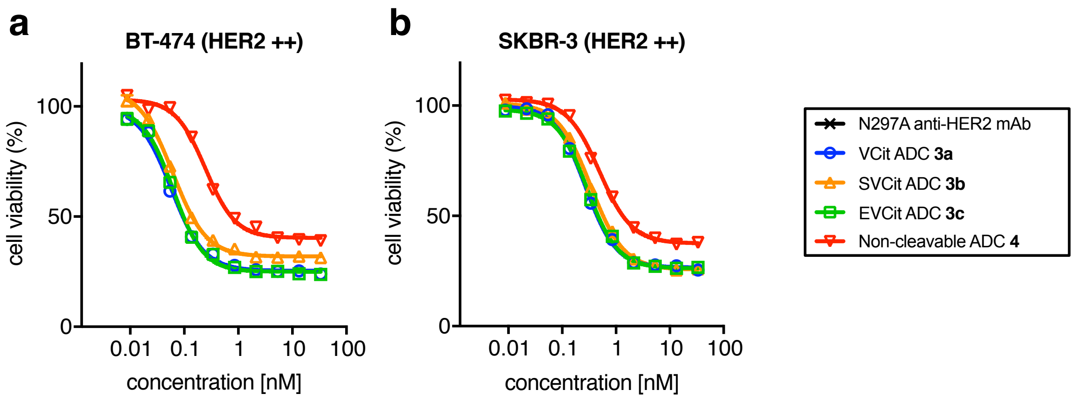

Supplementary Figure 11 In vitro cytotoxicity of ADCs. We tested unconjugated N297A anti-HER2 mAb (black), VCit ADC **3a** (blue), SVCit ADC **3b** (orange), EVCit ADC **3c** (green), and non-cleavable ADC **4** (red) using (**a**) BT-474 and (**b**) SKBR-3 cells. All assays were performed in quadruplicate. Error bars represent s.e.m.

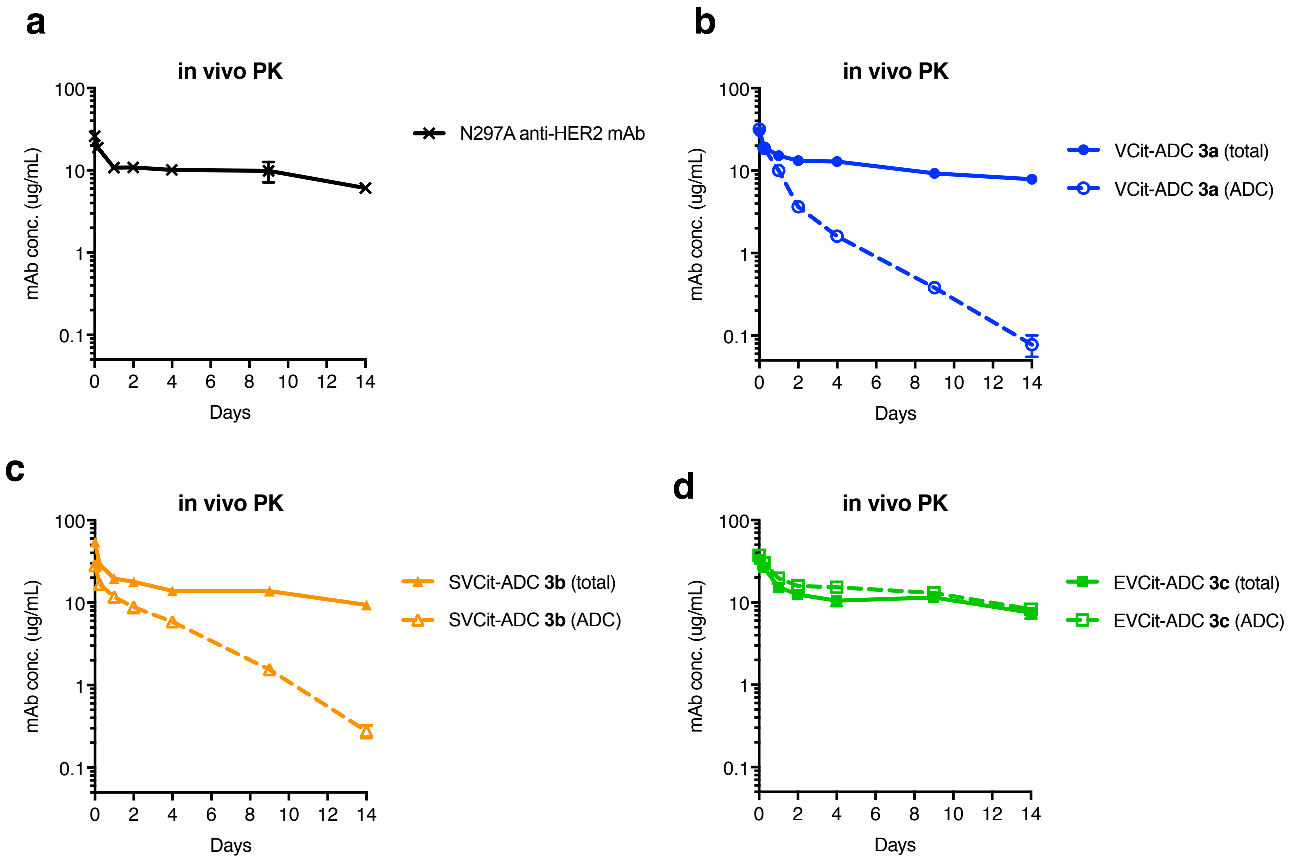

Supplementary Figure 12 In vivo pharmacokinetics (PK) of ADCs **3a**–**c**. (**a**) unmodified N297A anti-HER2 mAb, (**b**) VCit ADC **3a**, (**c**) SVCit ADC **3b**, and (**d**) EVCit ADC **3c** in female BALB/c mice (n = 3 per group). At the indicated time points, blood was collected to quantify concentrations of total antibody (conjugated and unconjugated, solid line) and ADC (conjugated only, dashed line) by sandwich ELISA. Error bars represent s.e.m.

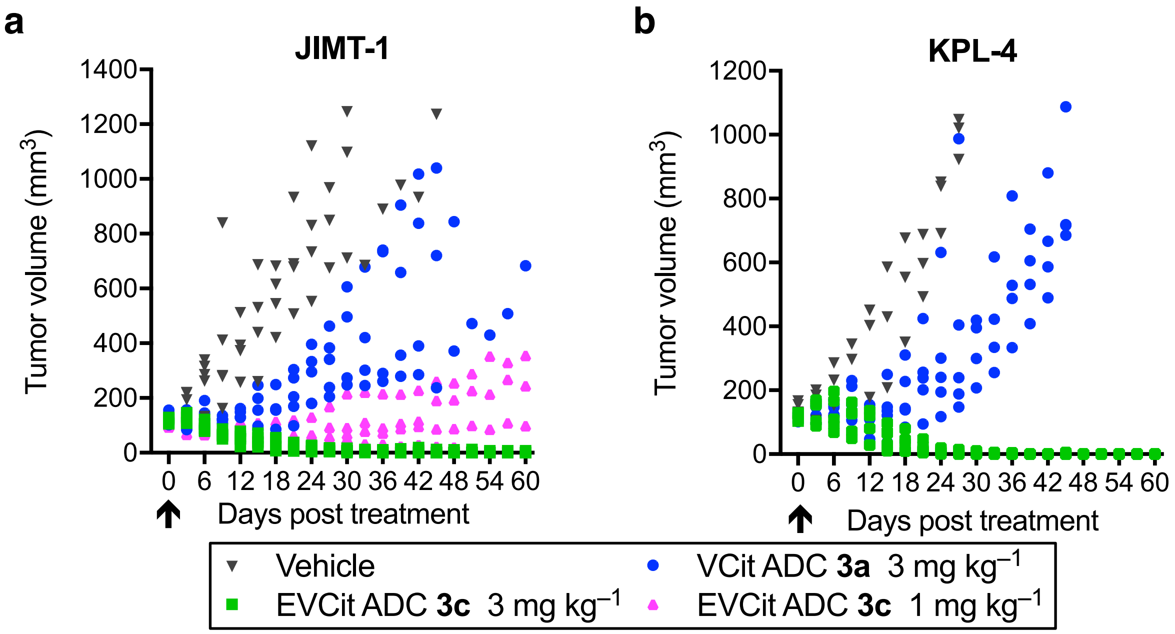

Supplementary Figure 13 Antitumor activity of anti-HER2 ADCs **3a**,**c**. The ADCs were tested in the (**a**) JIMT-1 and (**b**) KPL-4 xenograft tumor models (6–8-week female NCr nude mice, n = 3 for vehicle in the KPL-4 model; n = 5 for vehicle control in the JIMT-1 model and ADCs in both models). Each dot represents the individual mouse treated with vehicle (gray), 3 mg kg^{-1} VCit ADC **3a** (blue), 3 mg kg^{-1} EVCit ADC **3c** (green), or 1 mg kg^{-1} **3c** (magenta, only in the JIMT-1 model). A single dose of each ADC (1 or 3 mg kg^{-1}) or vehicle was administered intravenously to mice when a mean tumor volume reached ~100 mm^3 (indicated with a black arrow). Tumor volume and body weight were monitored every 3 days. Mice were euthanized when the tumor volume exceeded 1000 mm^3, the tumor size exceeded 2 cm in diameter, greater than 15% weight loss was observed, or mice showed signs of distress.

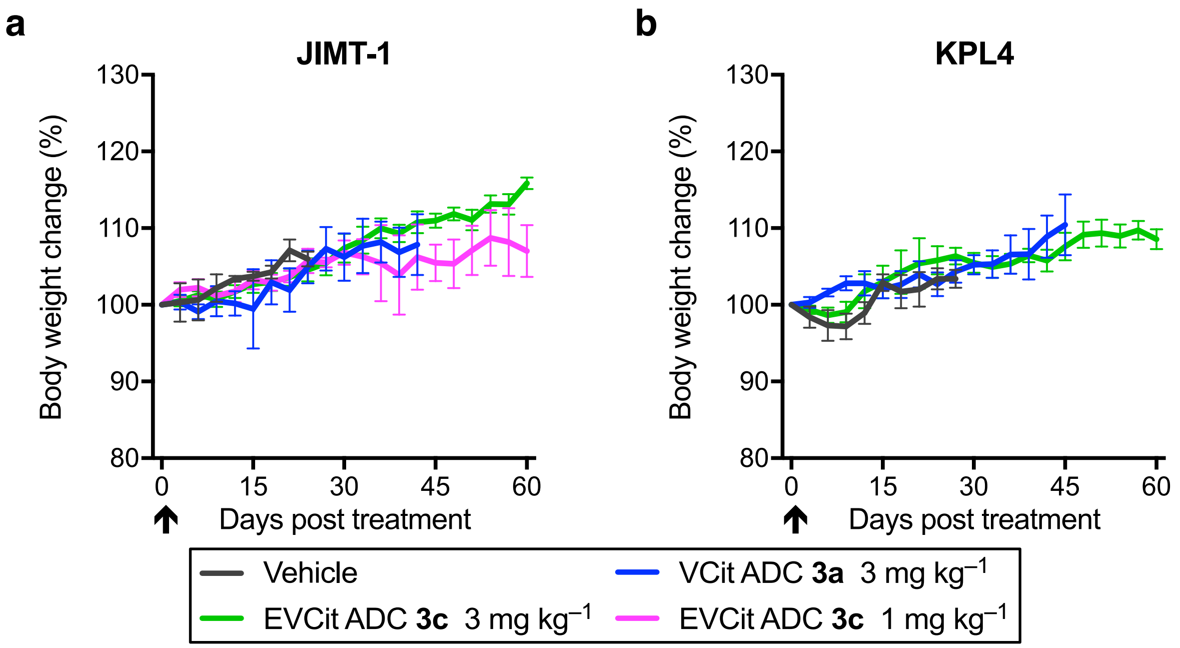

Supplementary Figure 14 Body weight change during treatment. Vehicle (gray), VCit ADC **3a** (blue), and EVCit ADC **3c** (3 mg kg^{-1} in green, 1 mg kg^{-1} in magenta) were injected to the (**a**) JIMT-1 and (**b**) KPL-4 xenograft tumor models (female NCr nude mice, n = 3 for vehicle in the KPL-4 model; n = 5 for vehicle in the JIMT-1 model and ADCs in both models). No significant body weight loss caused by either ADC was observed over the course of study. Error bars represent s.e.m.

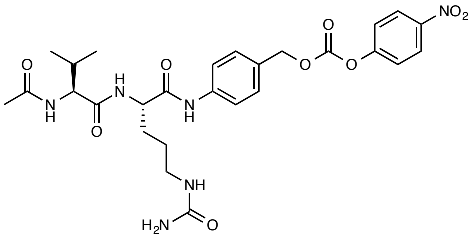

S2a

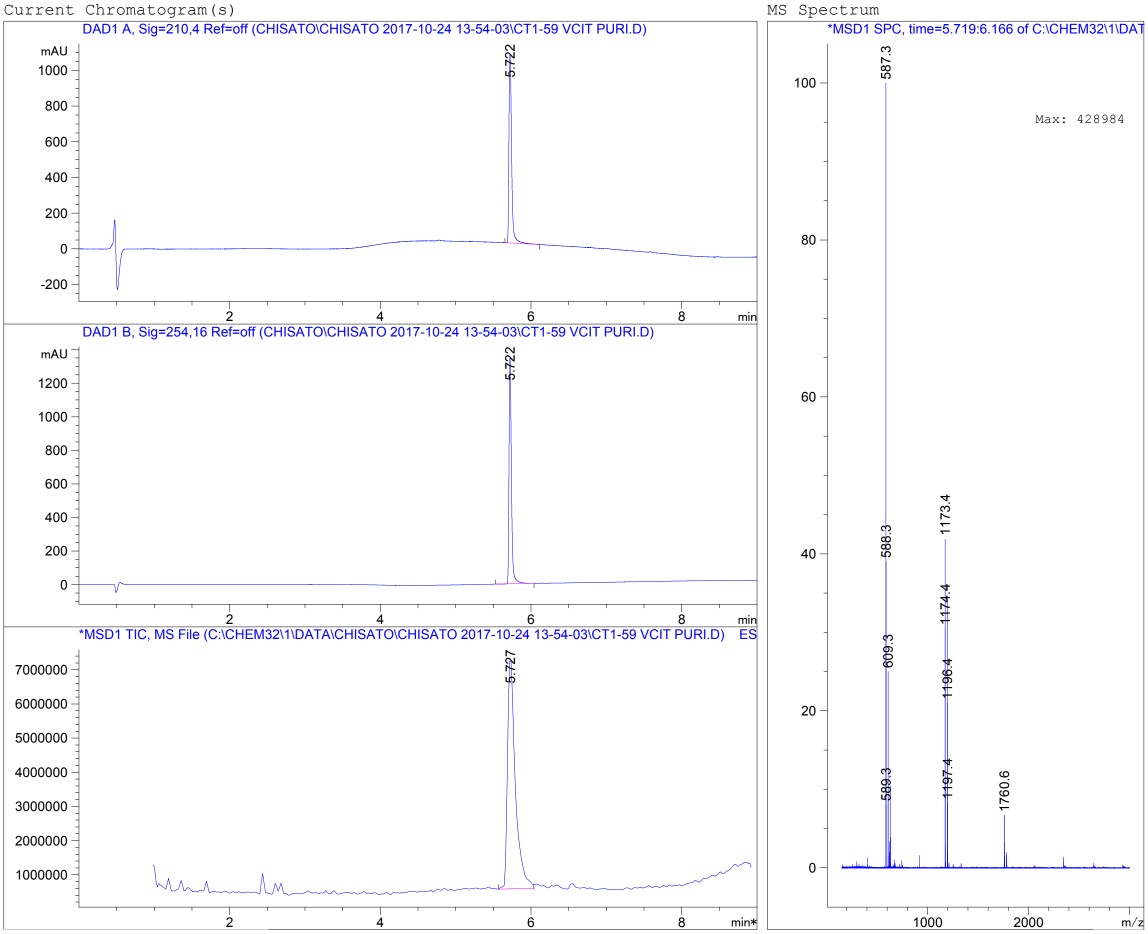

Supplementary Figure 15 HPLC trace and low-resolution ESI-MS spectrum of pure product **S2a**.

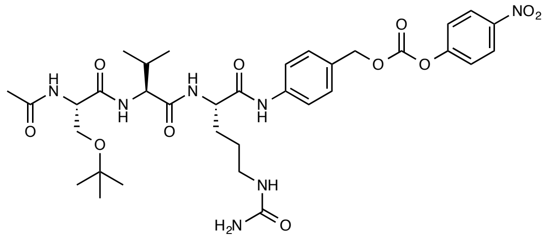

S2b

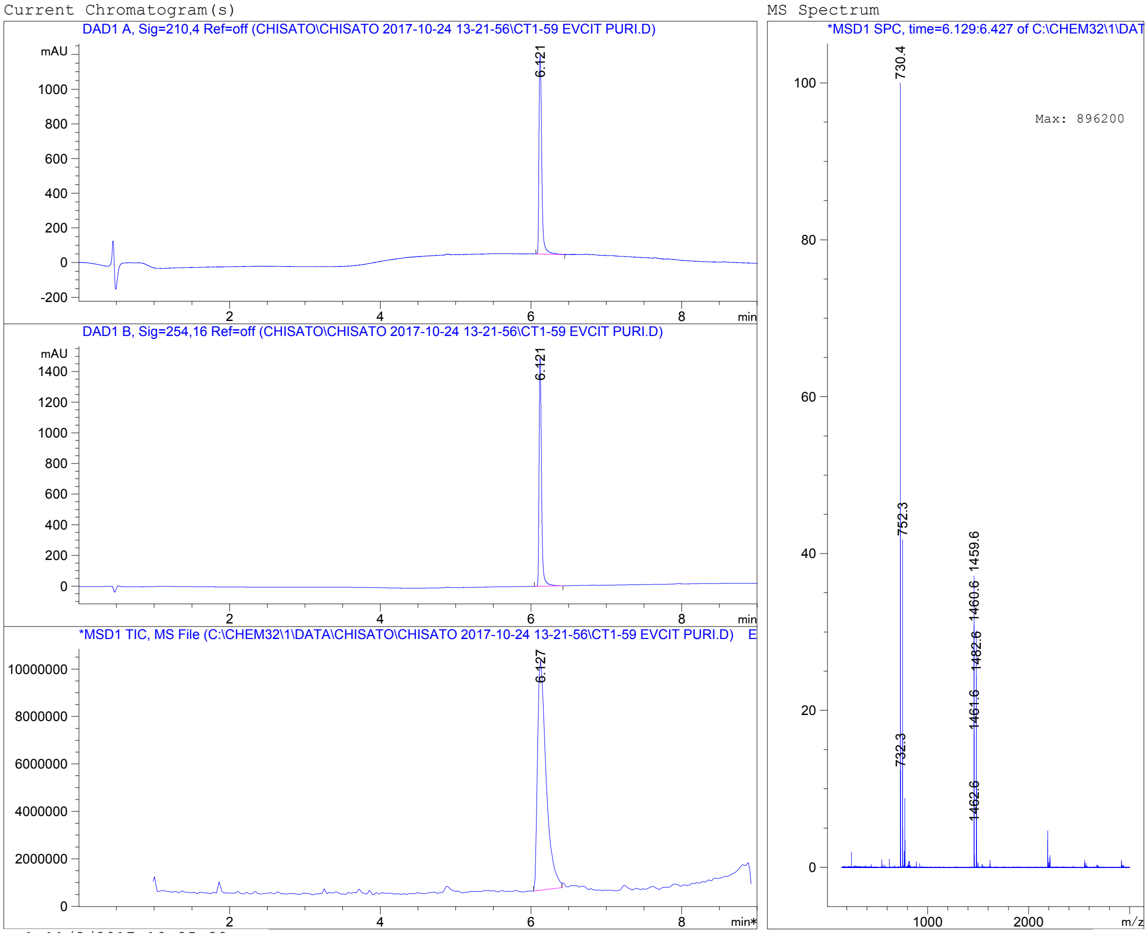

Supplementary Figure 16 HPLC trace and low-resolution ESI-MS spectrum of pure product **S2b**.

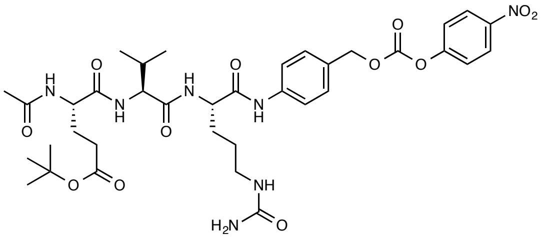

S2c

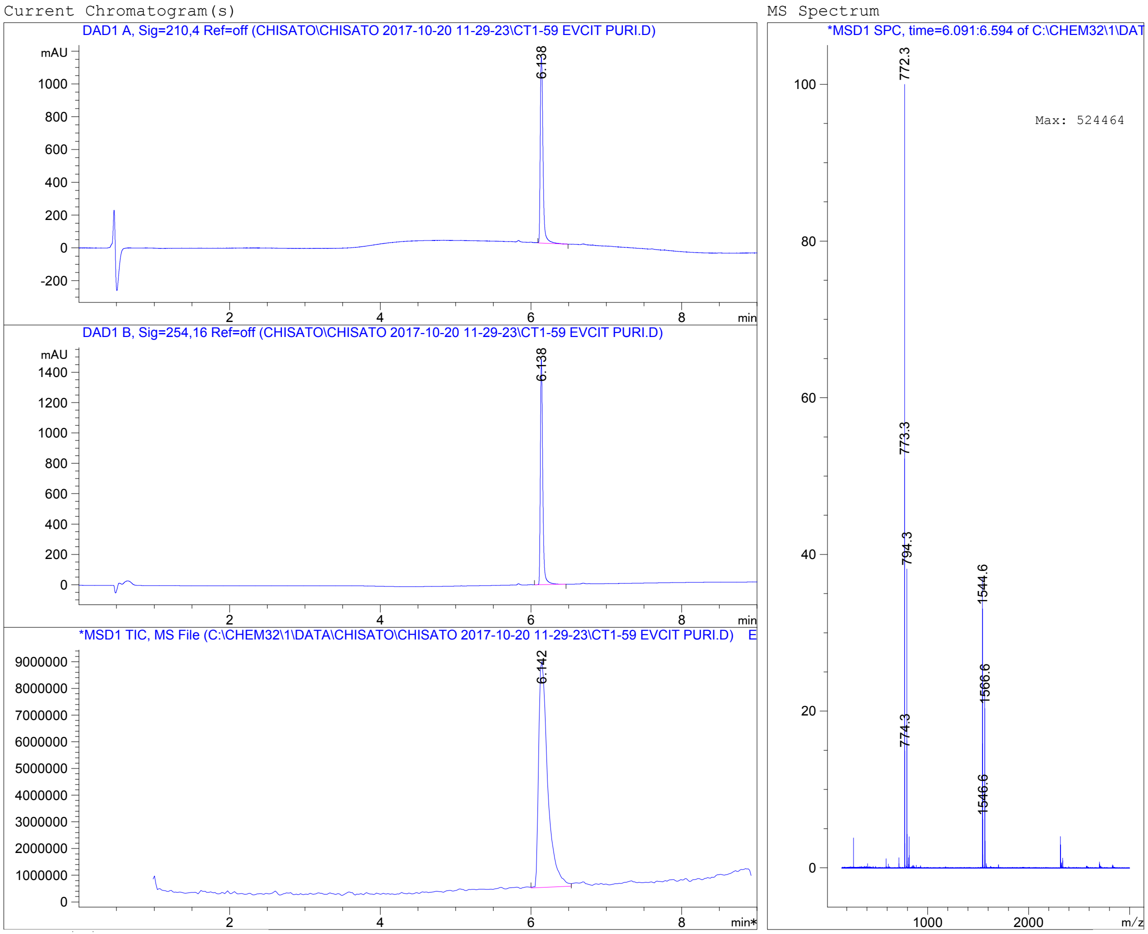

Supplementary Figure 17 HPLC trace and low-resolution ESI-MS spectrum of pure product **S2c**.

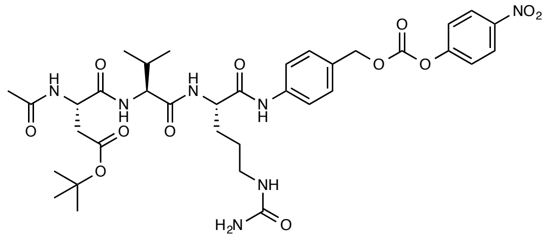

S2d

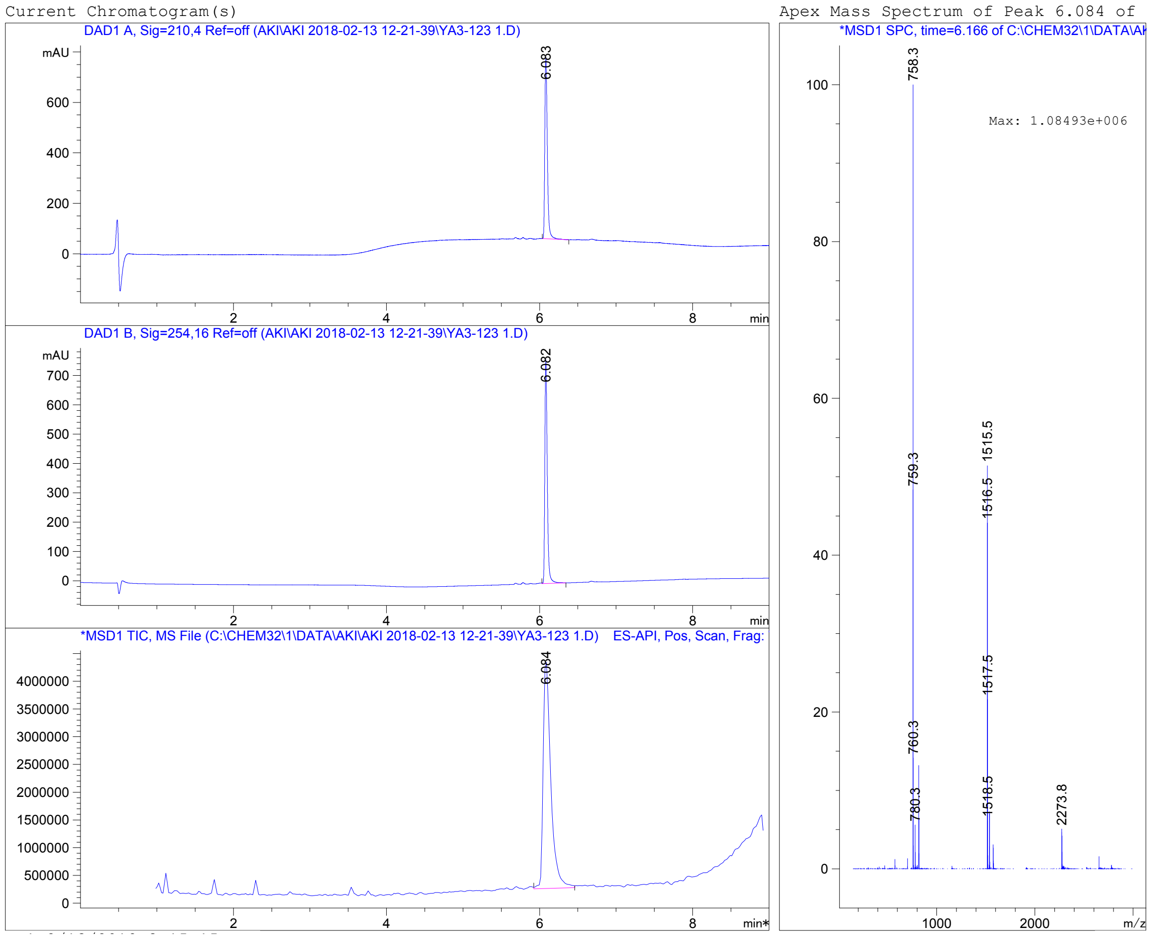

Supplementary Figure 18 HPLC trace and low-resolution ESI-MS spectrum of pure product **S2d**.

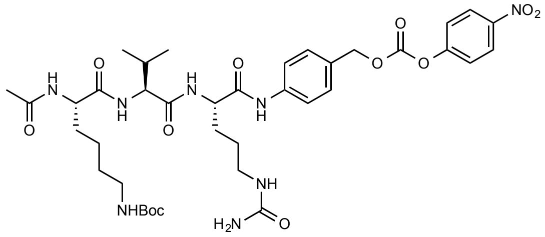

S2e

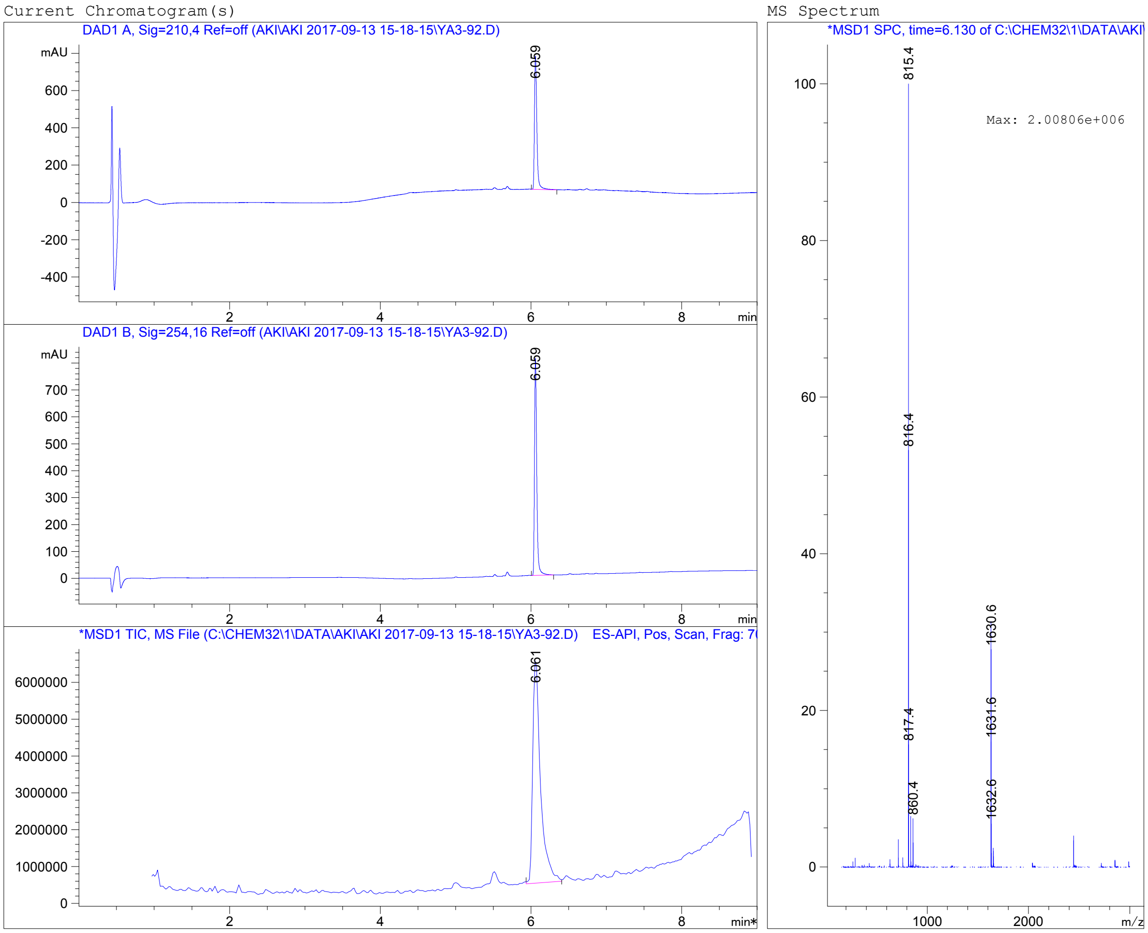

Supplementary Figure 19 HPLC trace and low-resolution ESI-MS spectrum of pure product **S2e**.

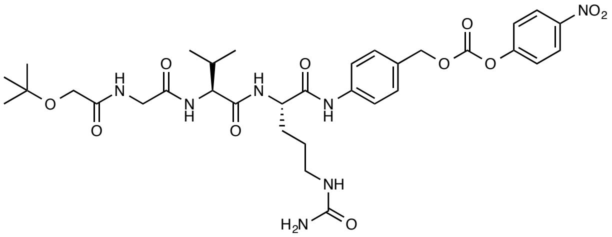

S2f

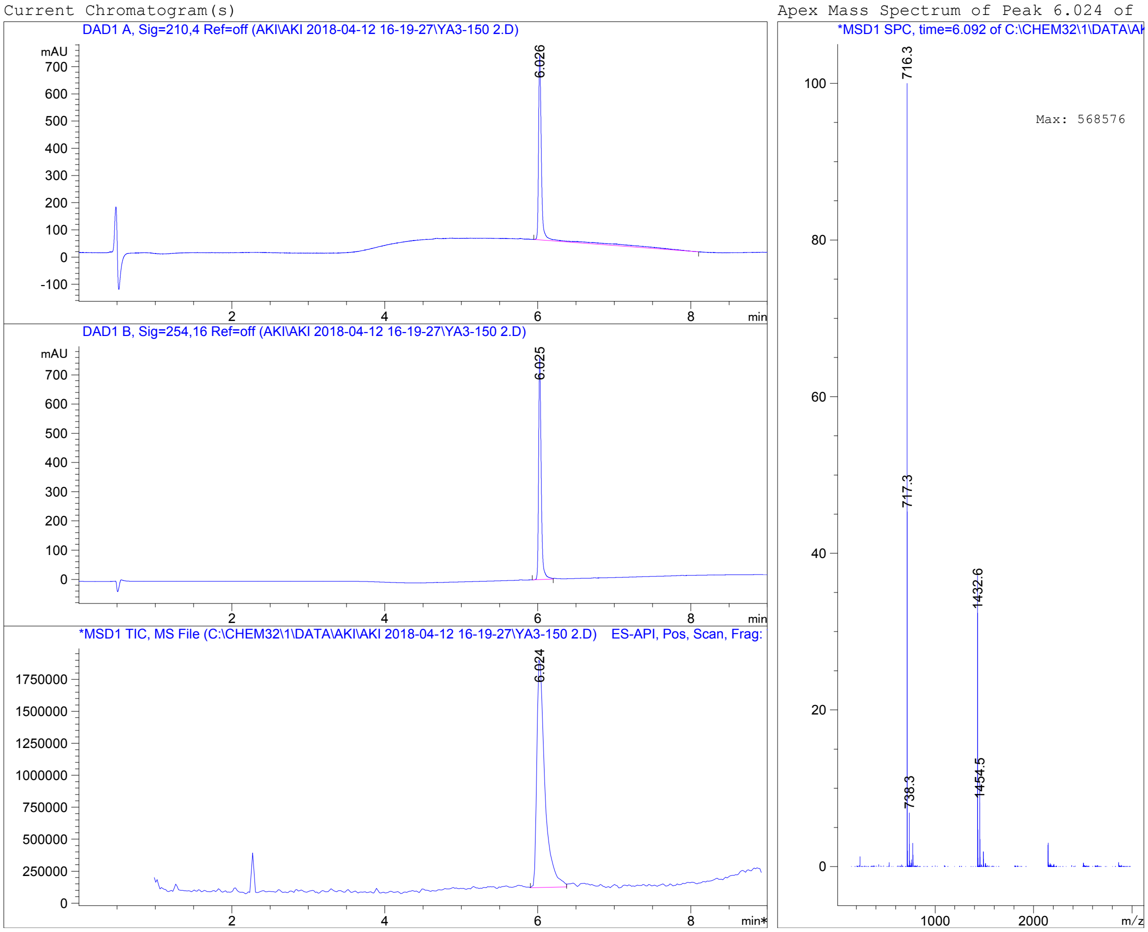

Supplementary Figure 20 HPLC trace and low-resolution ESI-MS spectrum of pure product **S2f**.

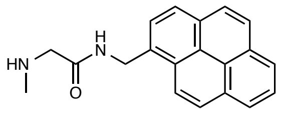

S3

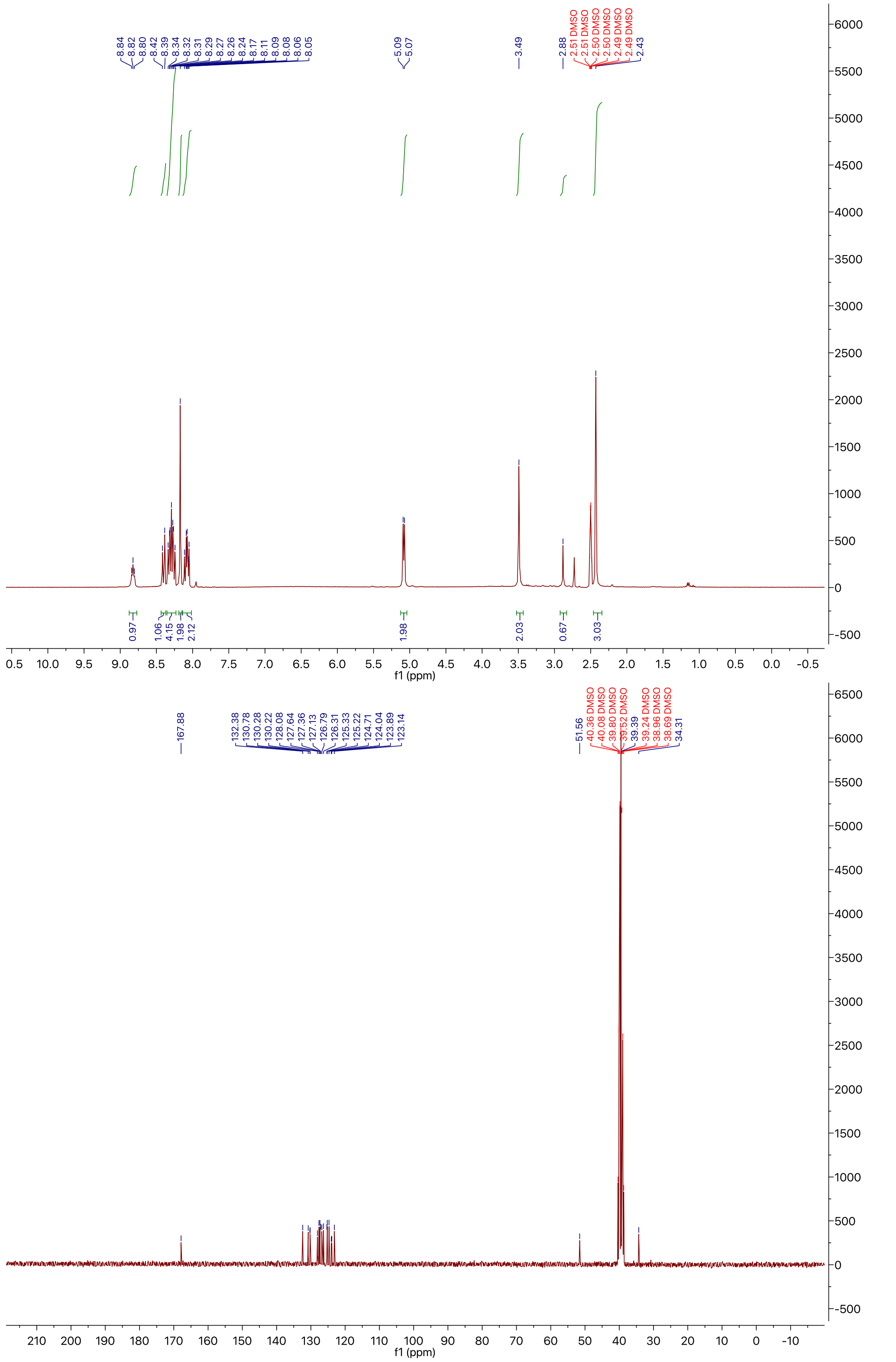

Supplementary Figure 21 NMR spectra of pure product **S3**. Top: ^{1}H-NMR; bottom: ^{13}C-NMR.

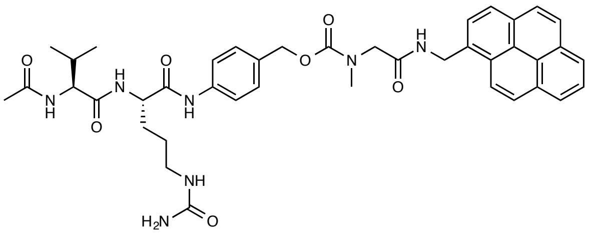

1a

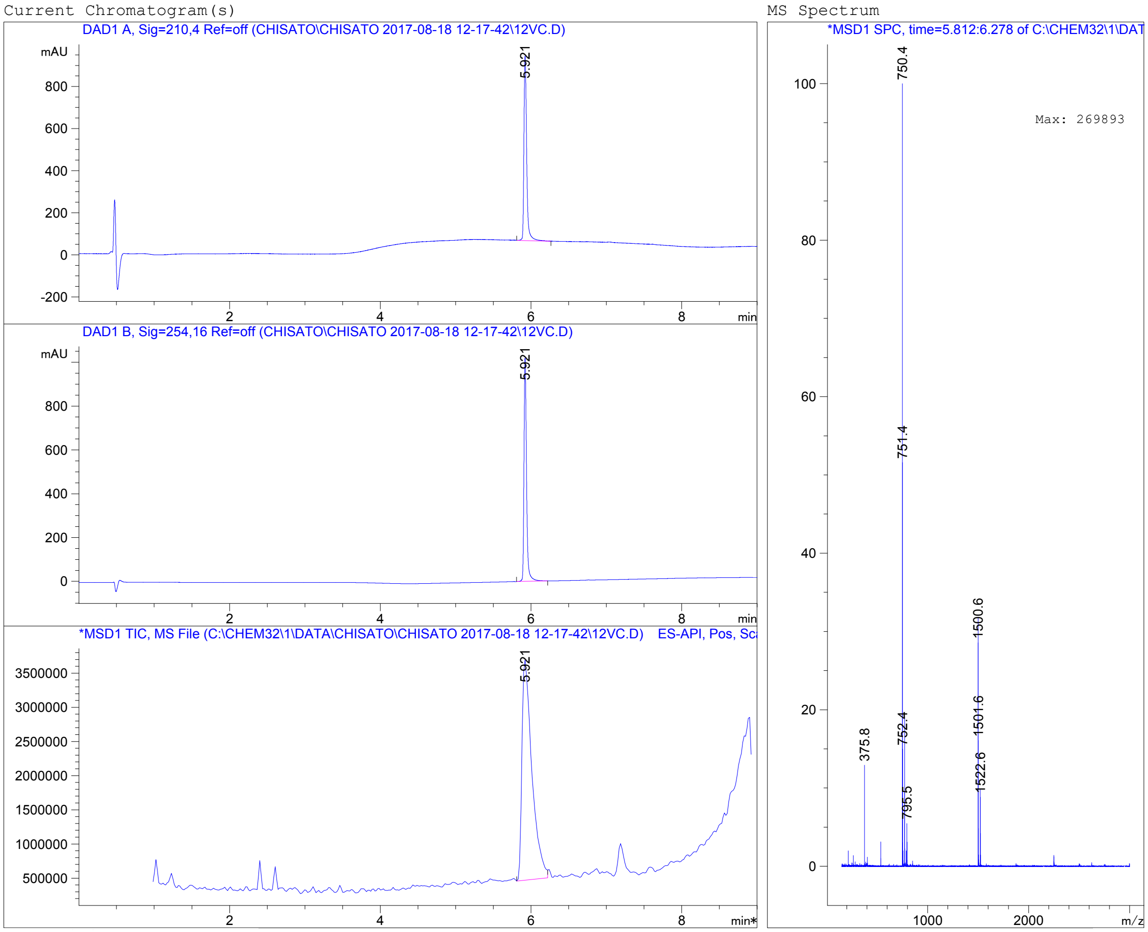

Supplementary Figure 22 HPLC trace and low-resolution ESI-MS spectrum of pure product **1a**.

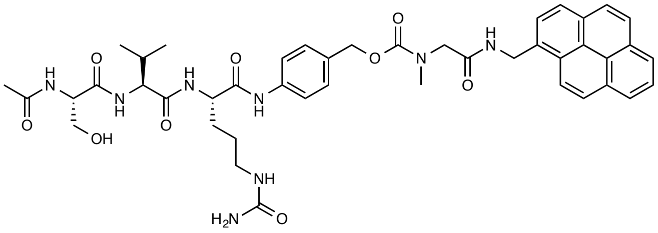

1b

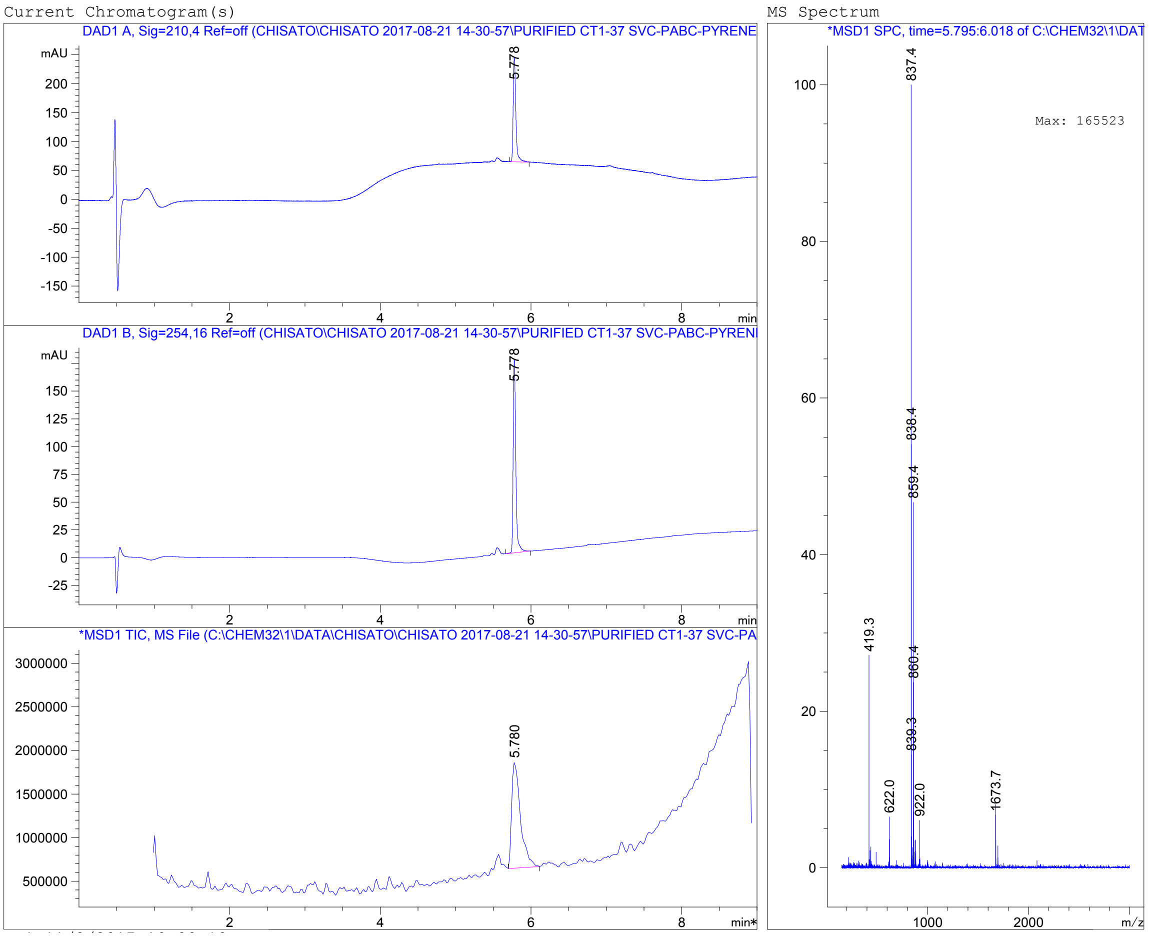

Supplementary Figure 23 HPLC trace and low-resolution ESI-MS spectrum of pure product **1b**.

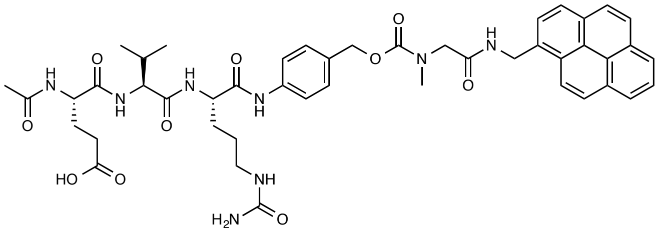

1c

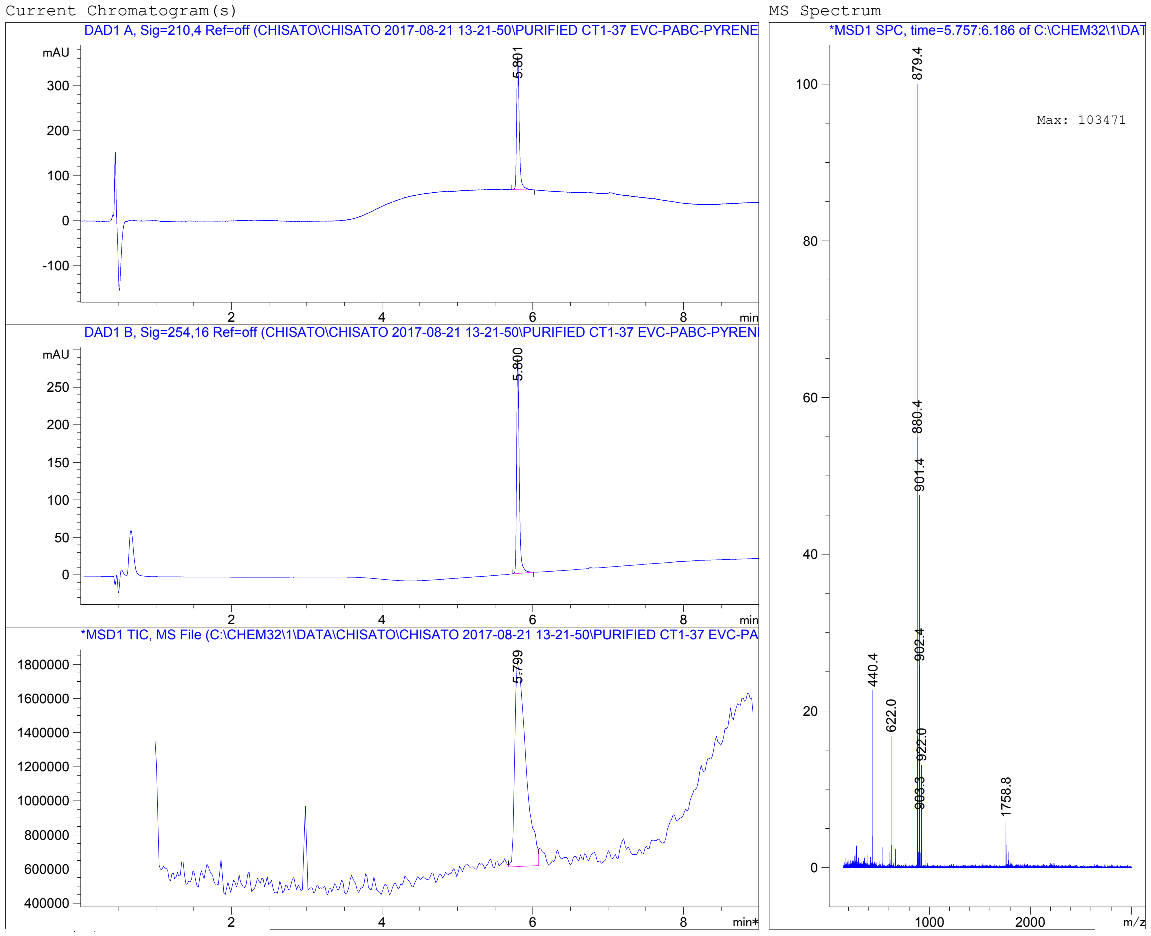

Supplementary Figure 24 HPLC trace and low-resolution ESI-MS spectrum of pure product **1c**.

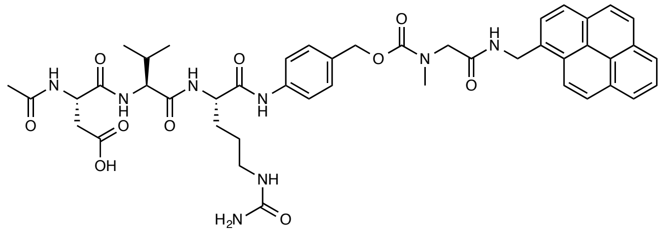

1d

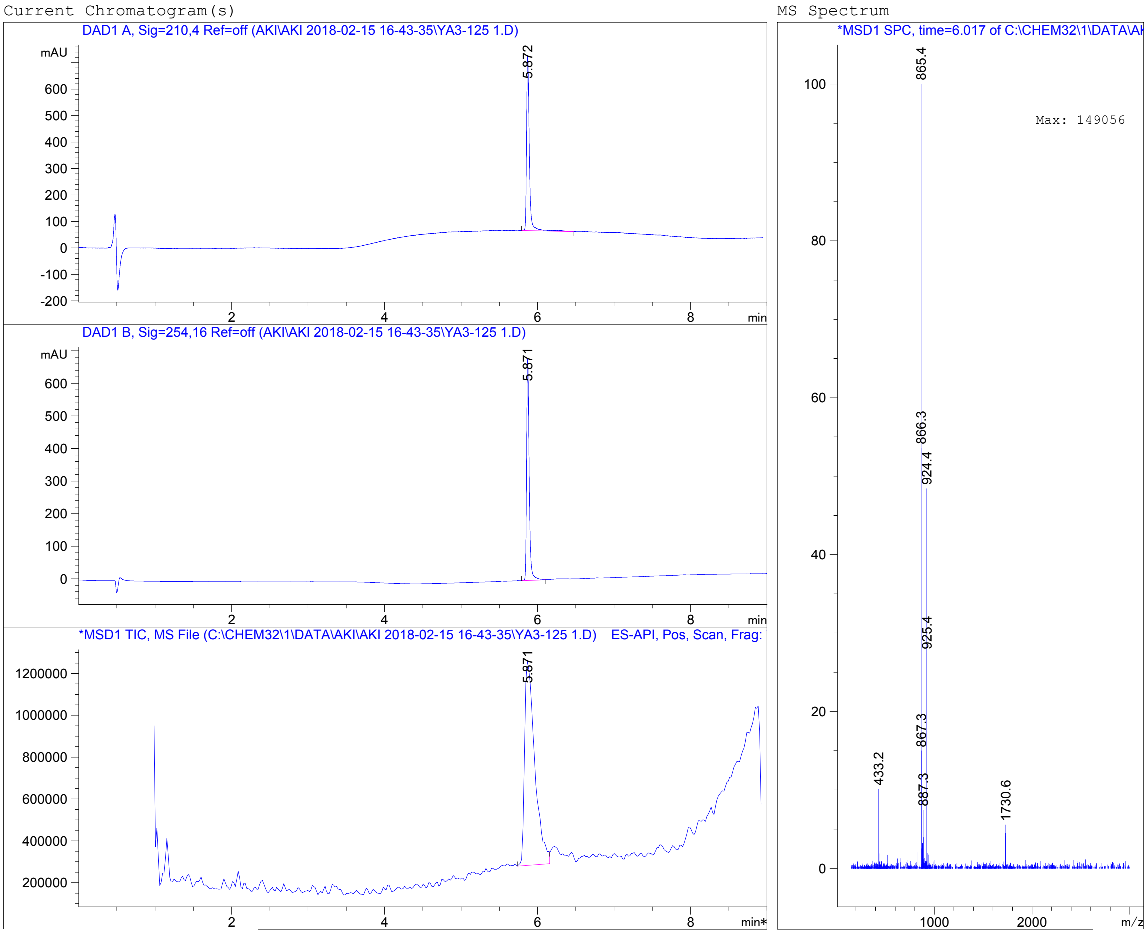

Supplementary Figure 25 HPLC trace and low-resolution ESI-MS spectrum of pure product **1d**.

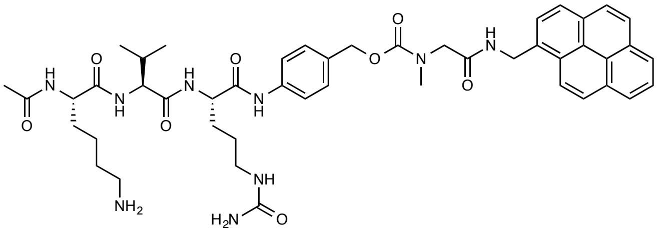

1e

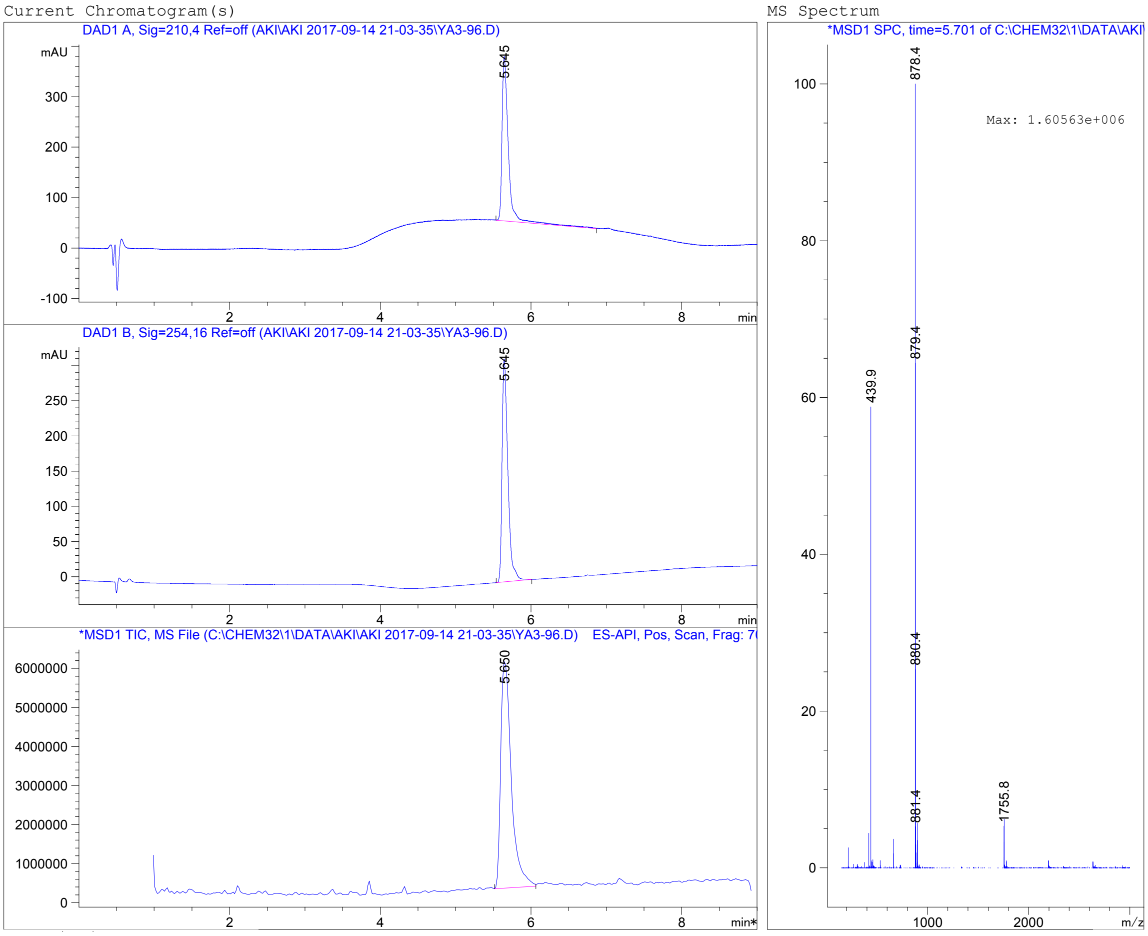

Supplementary Figure 26 HPLC trace and low-resolution ESI-MS spectrum of pure product **1e**.

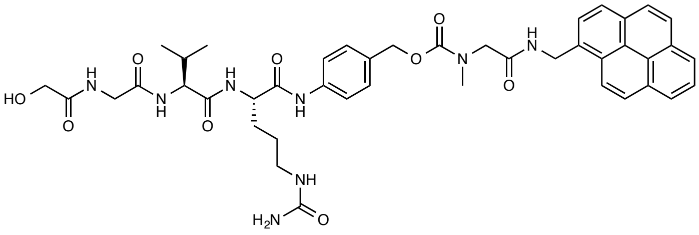

1f

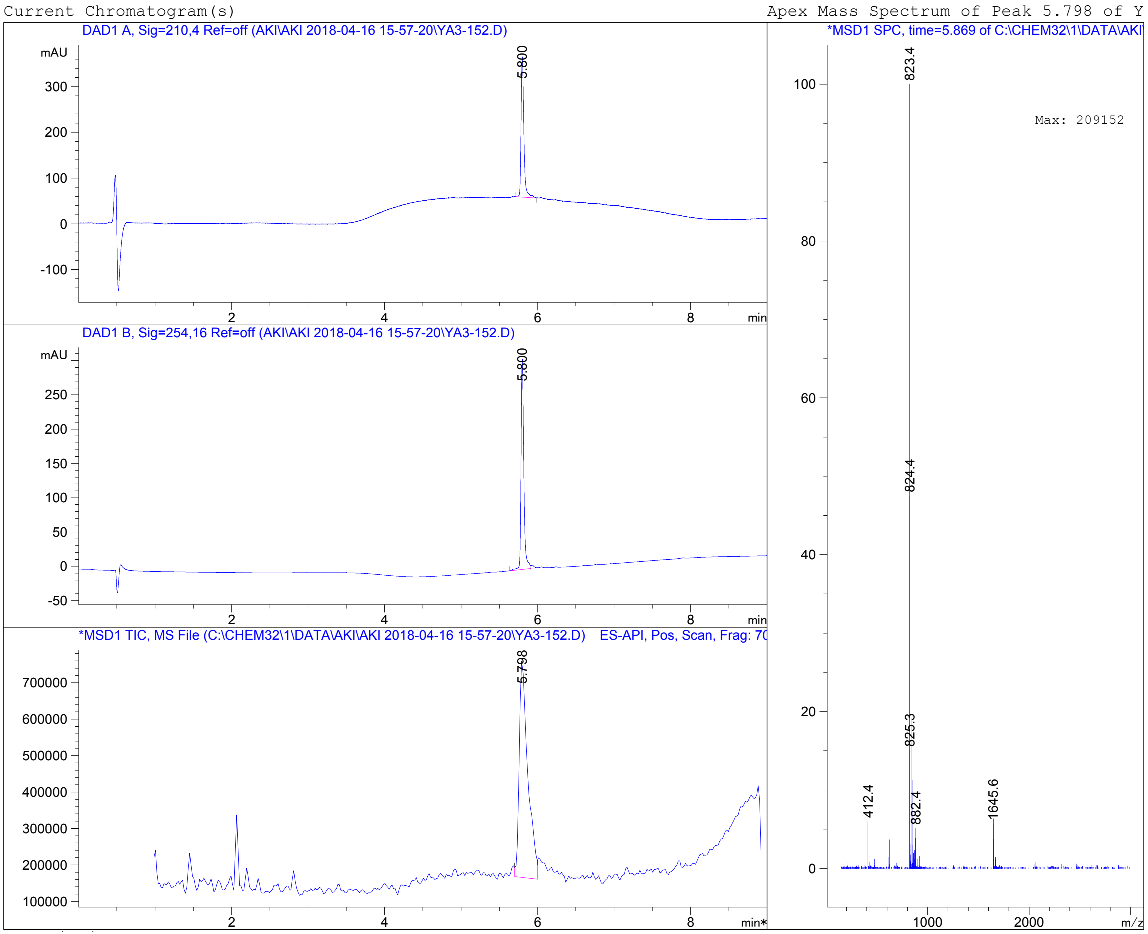

Supplementary Figure 27 HPLC trace and low-resolution ESI-MS spectrum of pure product **1f**.

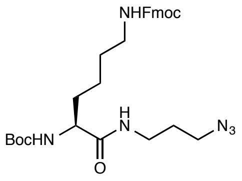

S4

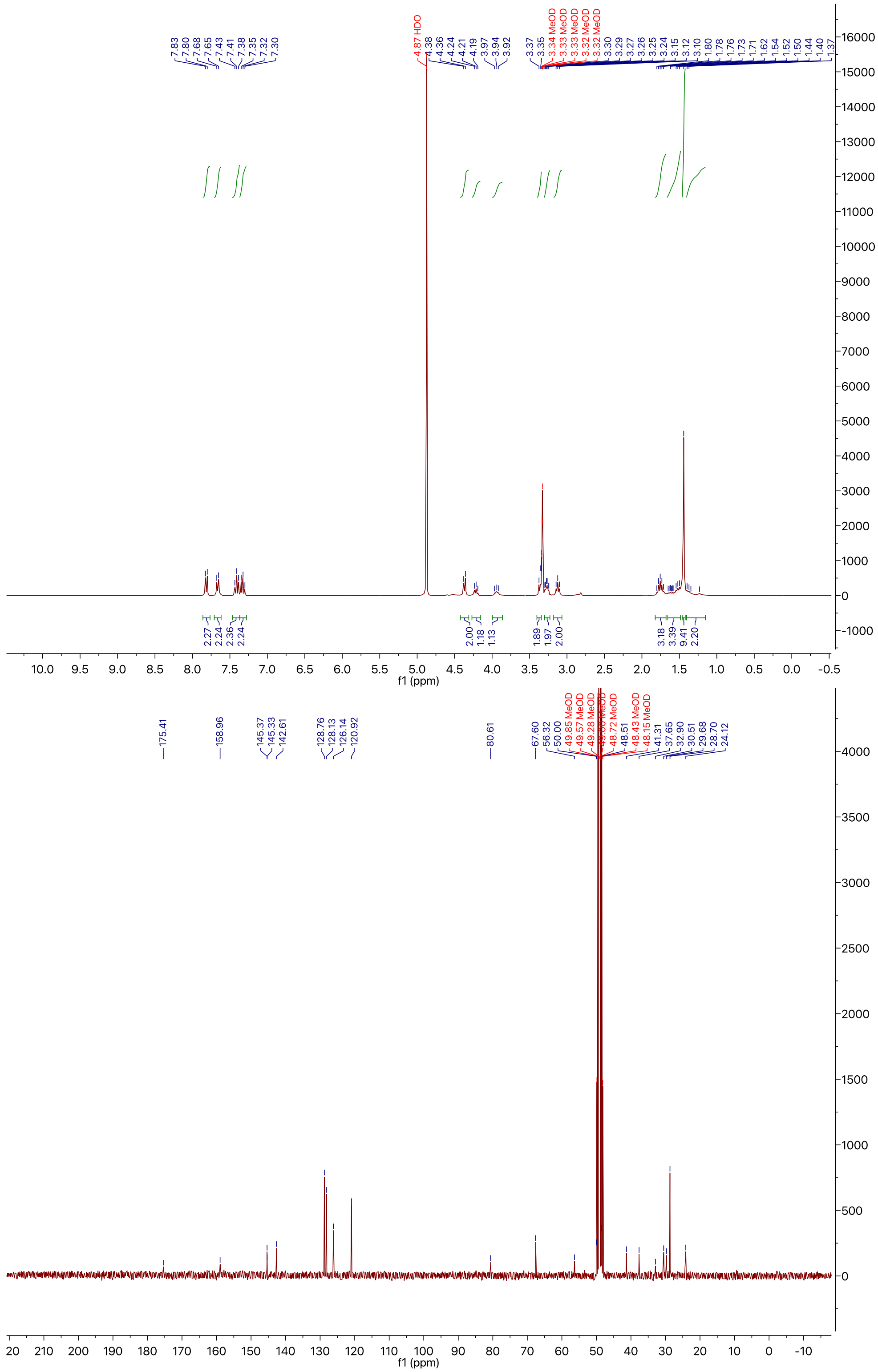

Supplementary Figure 28 NMR spectra of pure product **S4**. Top: ^{1}H-NMR; bottom: ^{13}C-NMR.

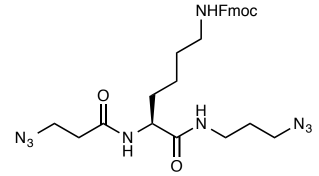

S5

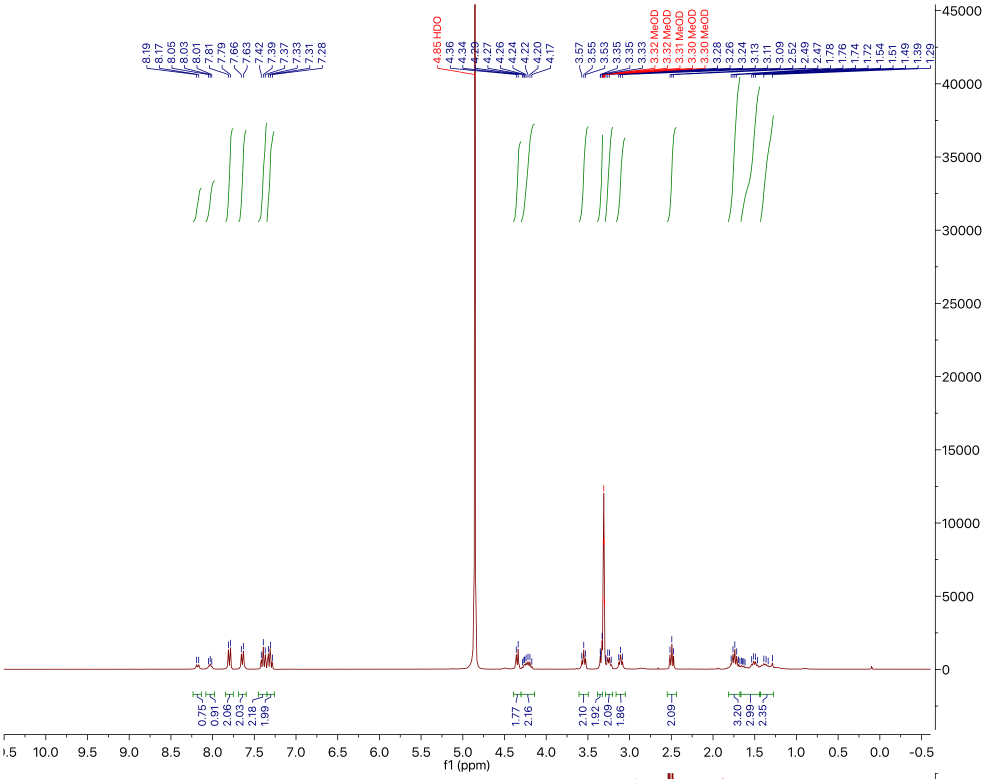

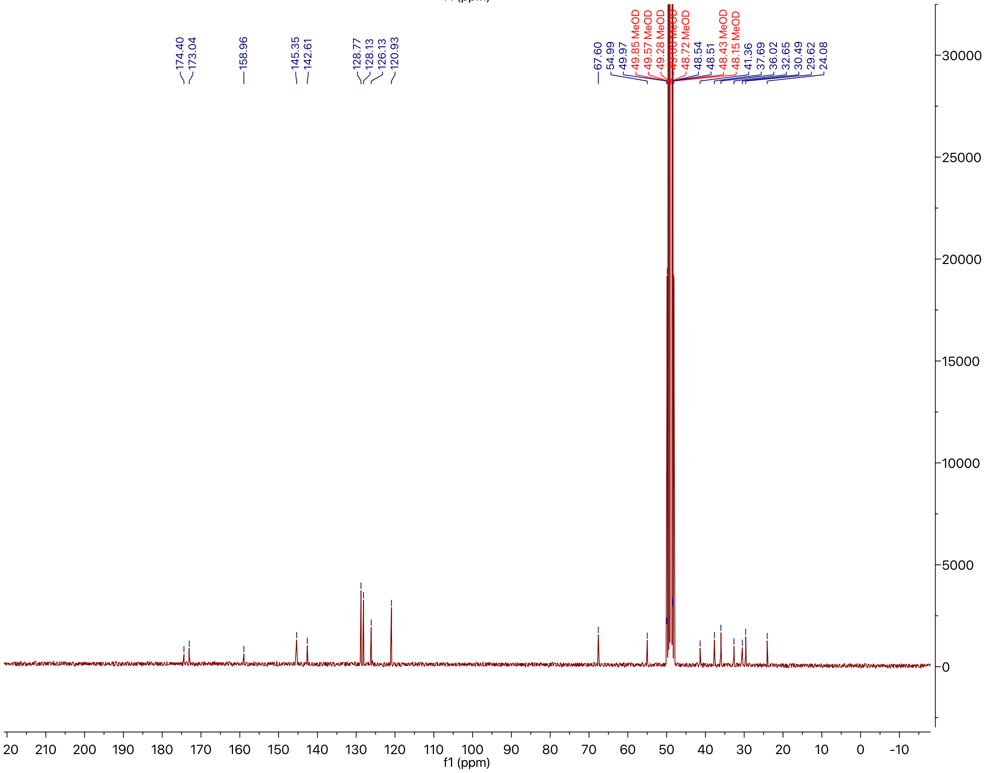

Supplementary Figure 29 NMR spectra of pure product **S5**. Top: ^{1}H-NMR; bottom: ^{13}C-NMR.

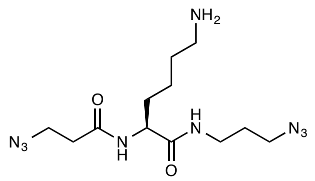

2

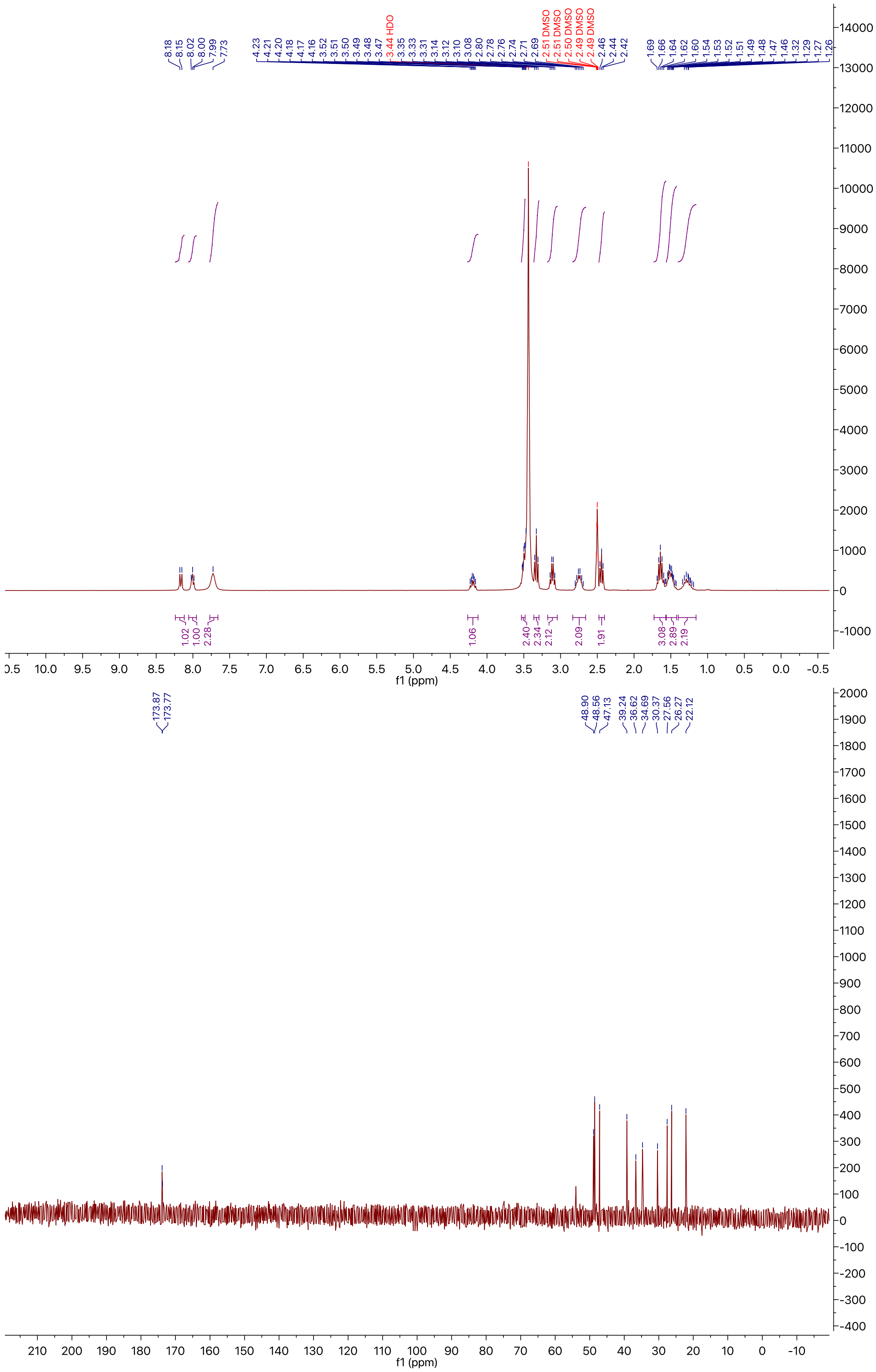

Supplementary Figure 30 NMR spectra of pure product **2**. Top: ^{1}H-NMR; bottom: ^{13}C-NMR.

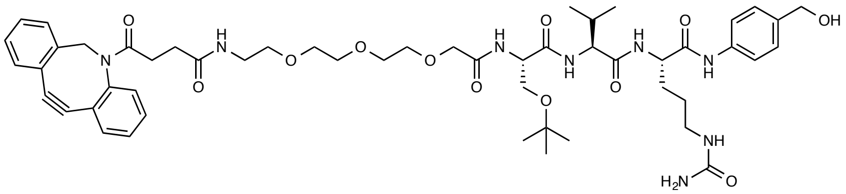

S6a

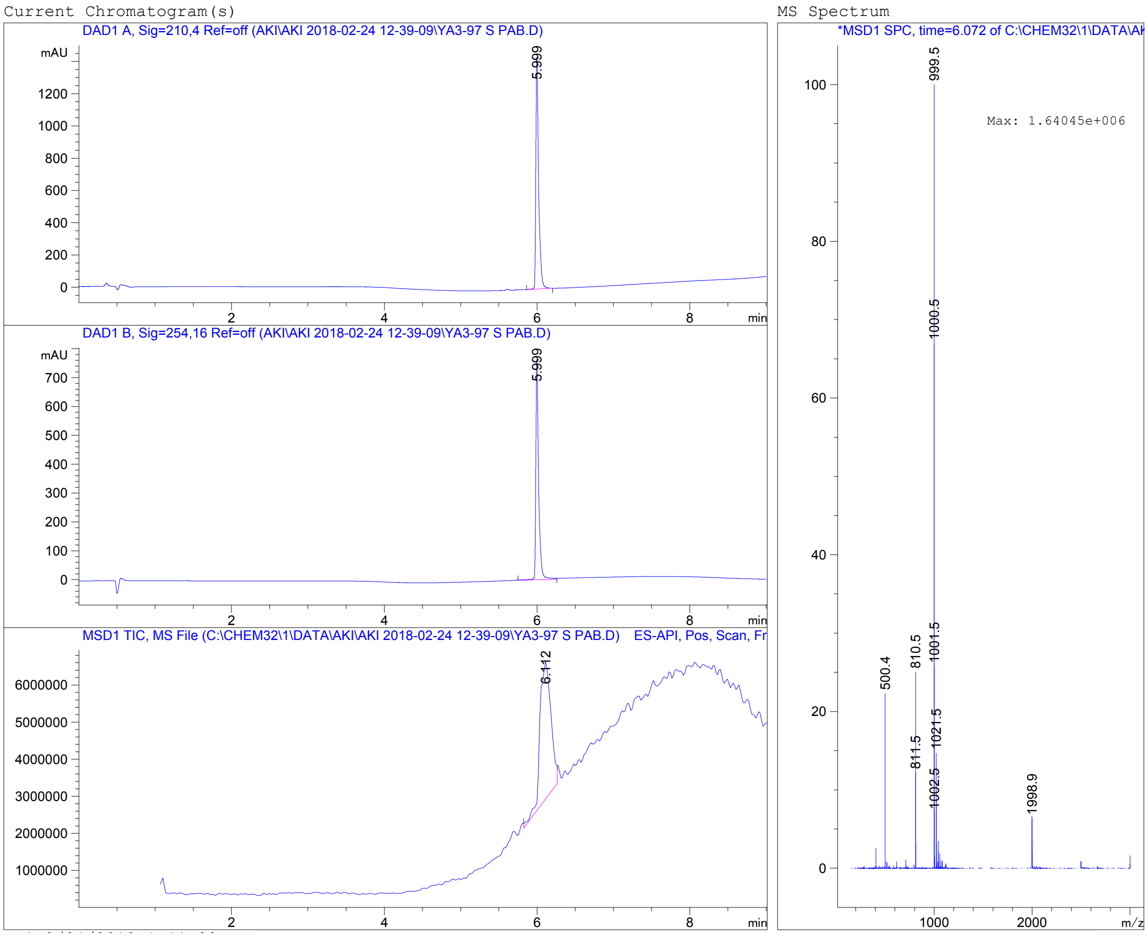

Supplementary Figure 31 HPLC trace and low-resolution ESI-MS spectrum of pure product **S6a**.

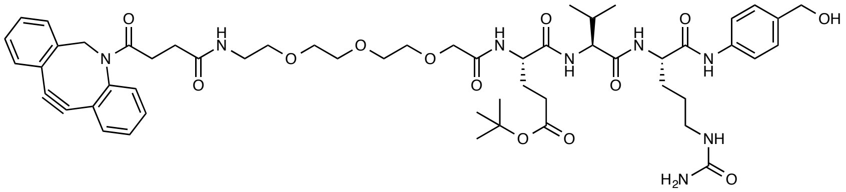

S6b

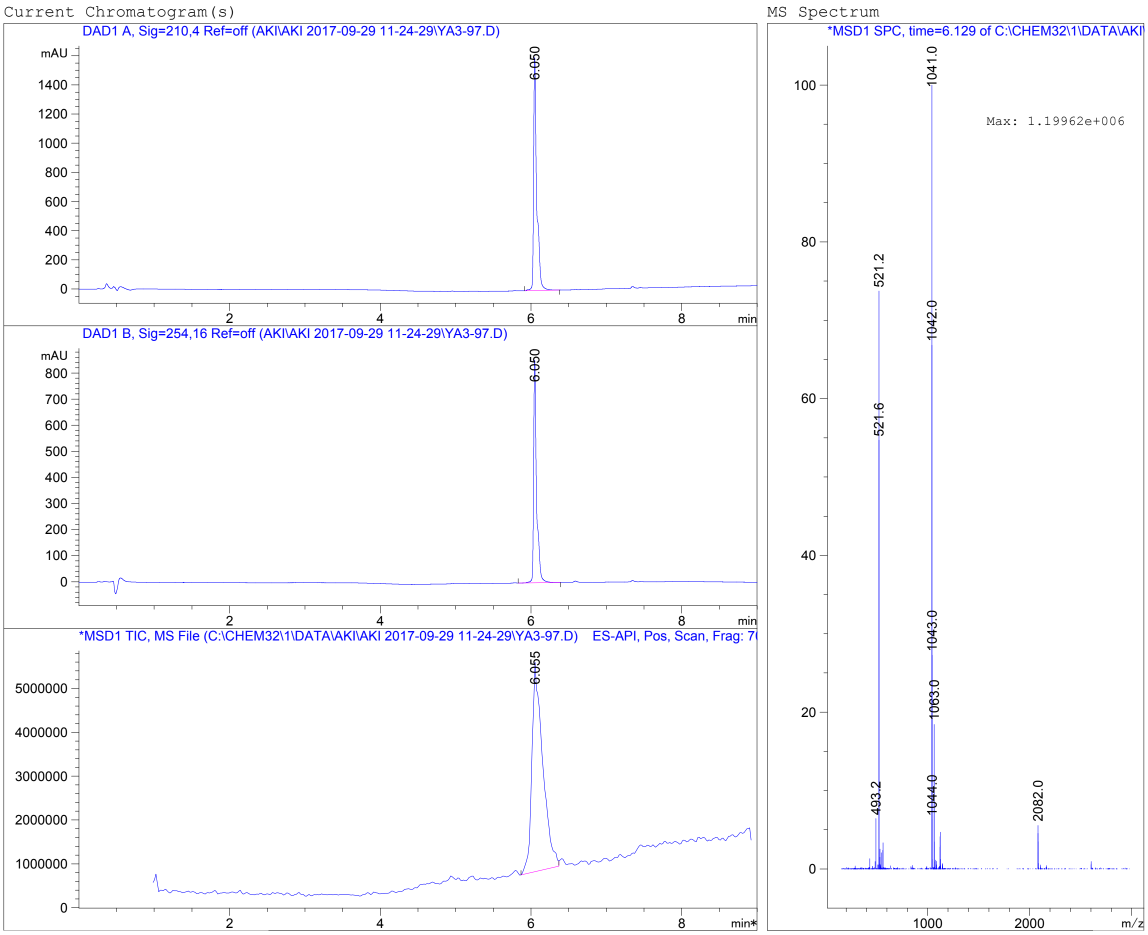

Supplementary Figure 32 HPLC trace and low-resolution ESI-MS spectrum of pure product **S6b**.

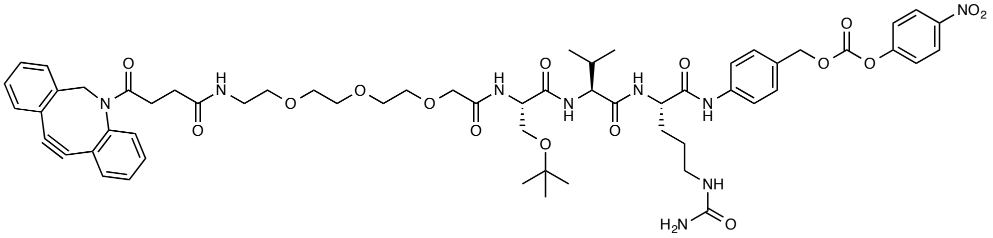

S7a

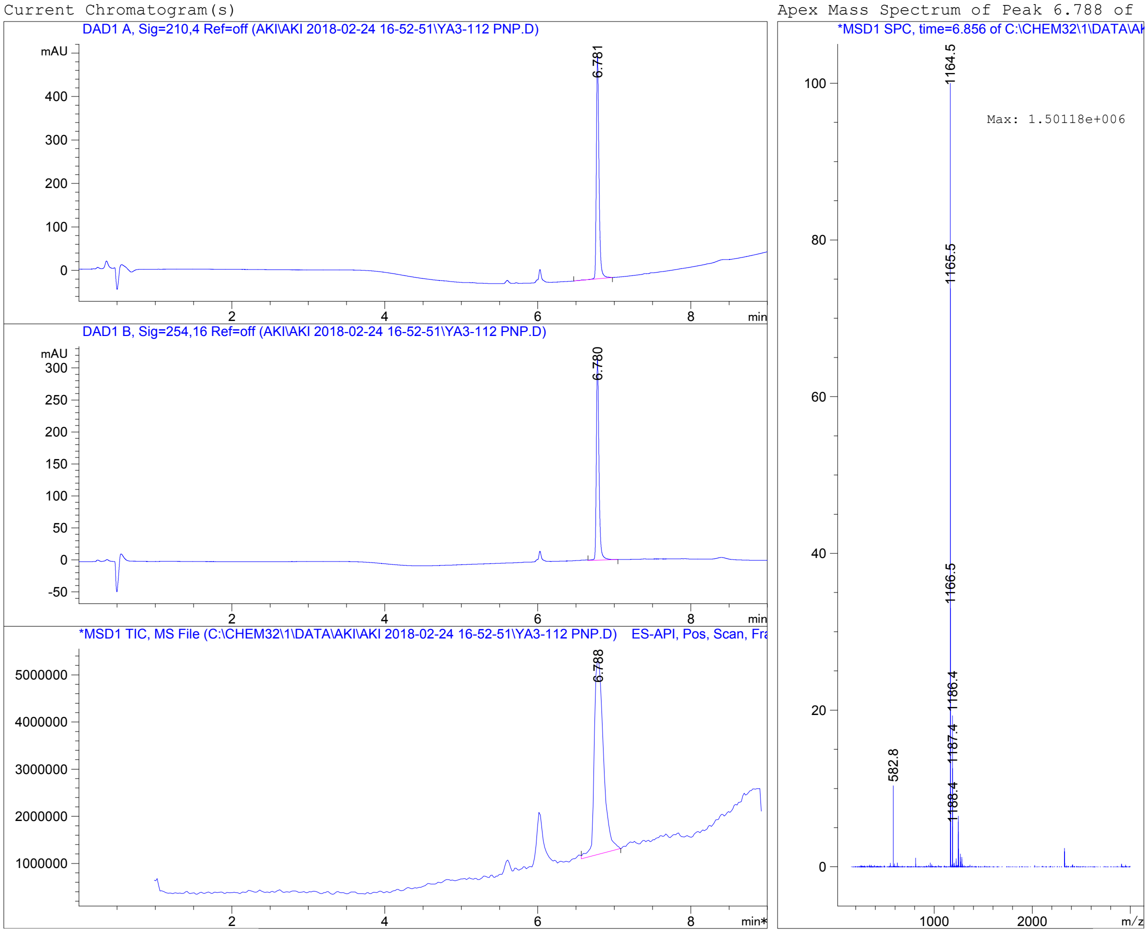

Supplementary Figure 33 HPLC trace and low-resolution ESI-MS spectrum of pure product **S7a**.

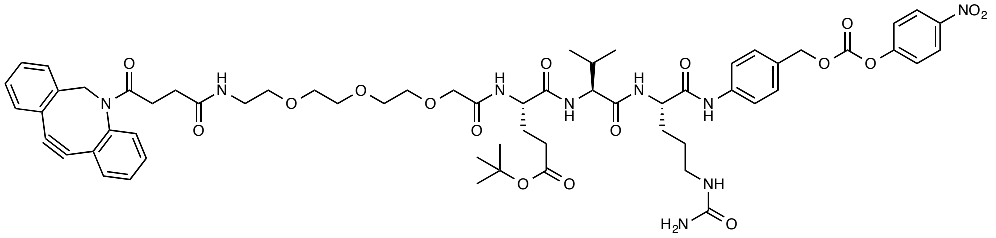

S7b

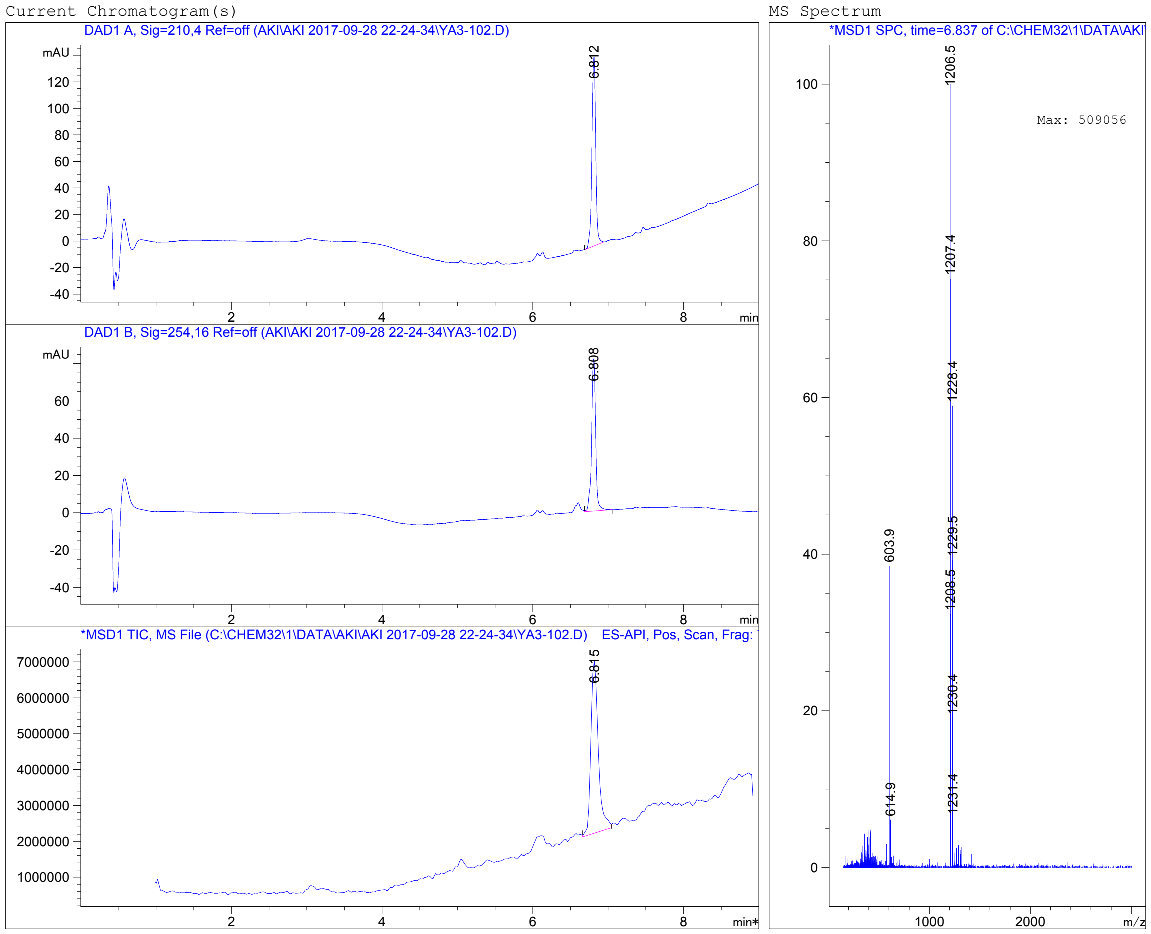

Supplementary Figure 34 HPLC trace and low-resolution ESI-MS spectrum of pure product **S7b**.

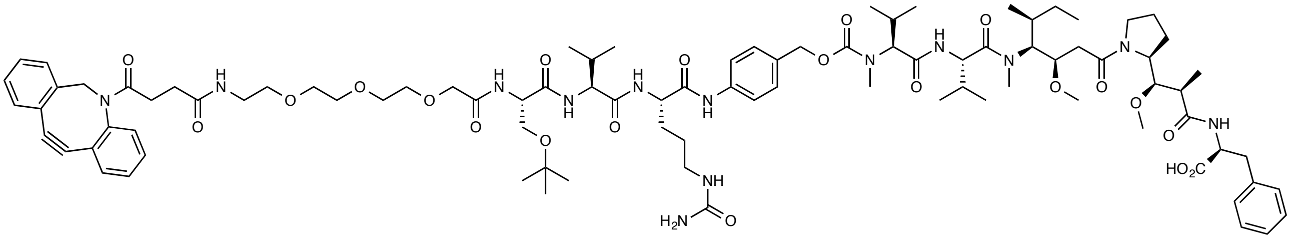

S8a

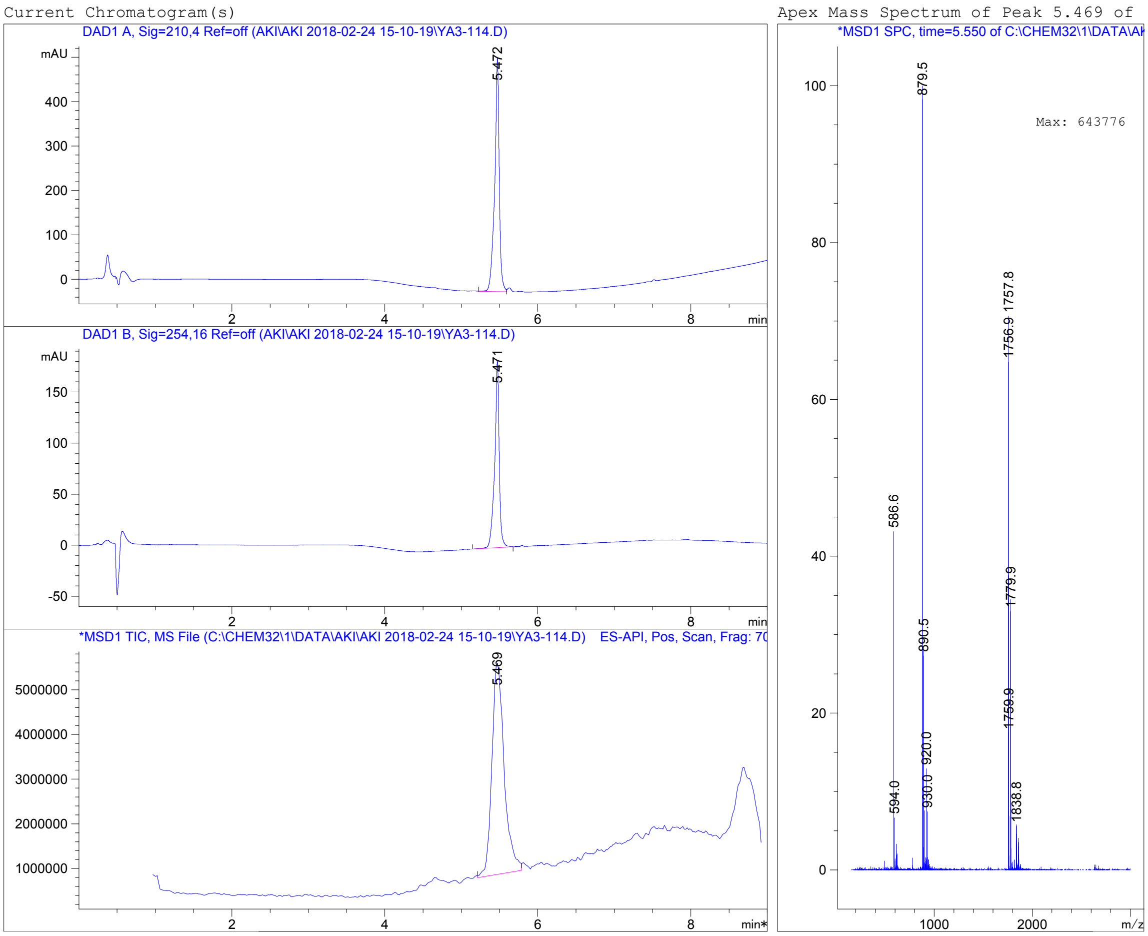

Supplementary Figure 35 HPLC trace and low-resolution ESI-MS spectrum of pure product **S8a**.

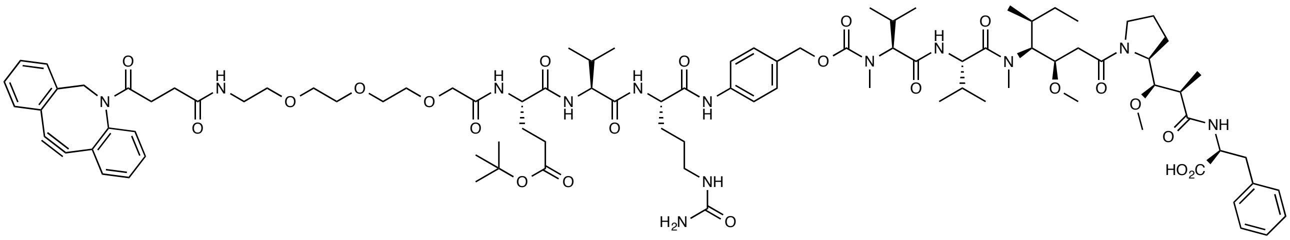

S8b

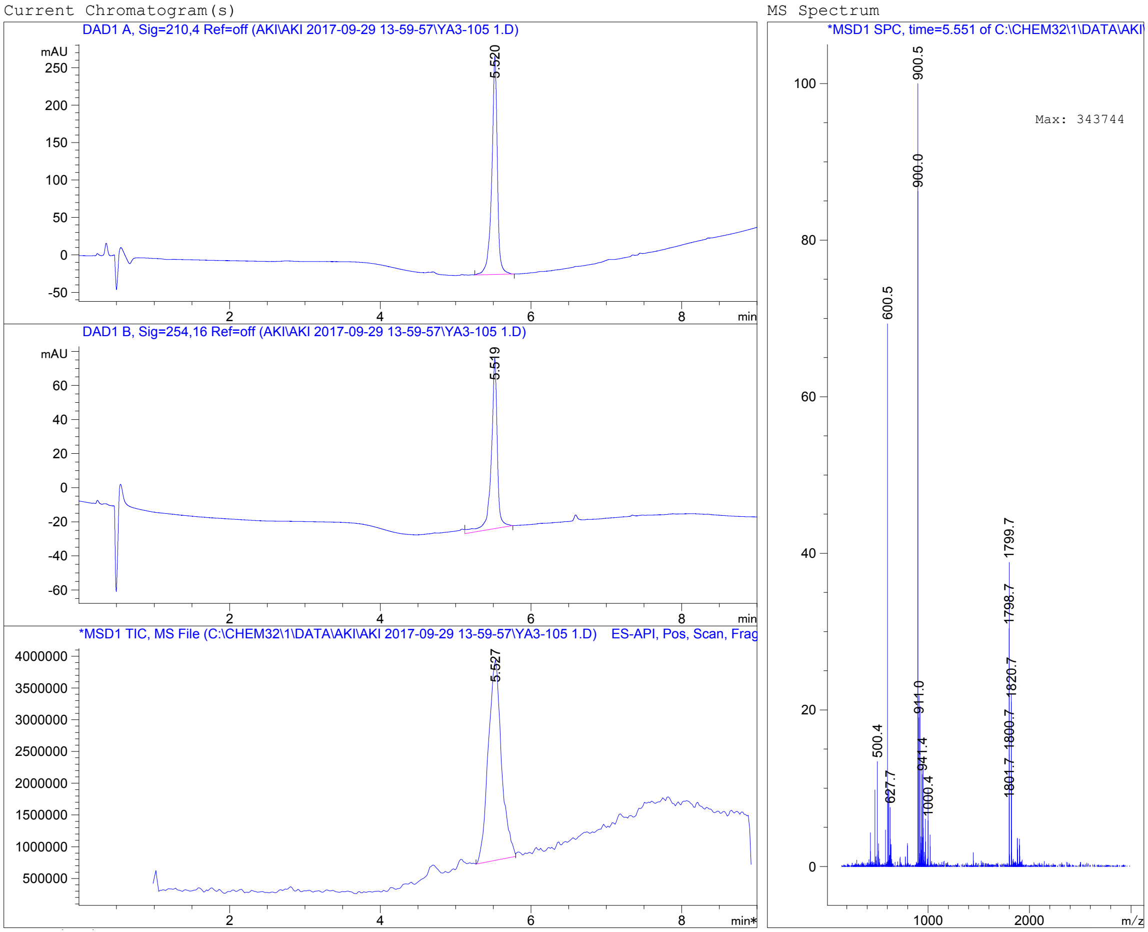

Supplementary Figure 36 HPLC trace and low-resolution ESI-MS spectrum of pure product **S8b**.

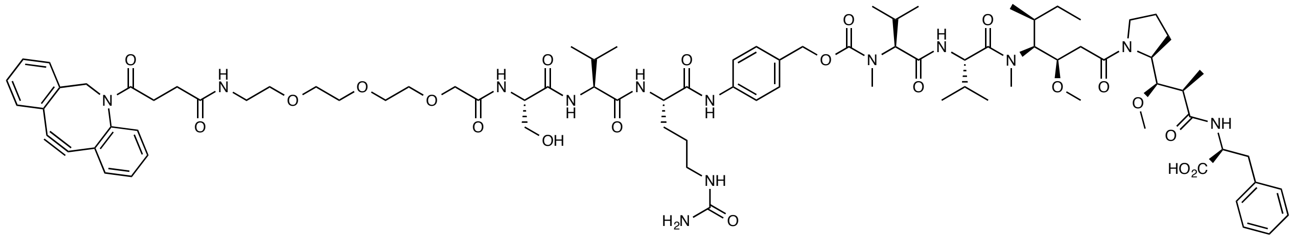

S9a

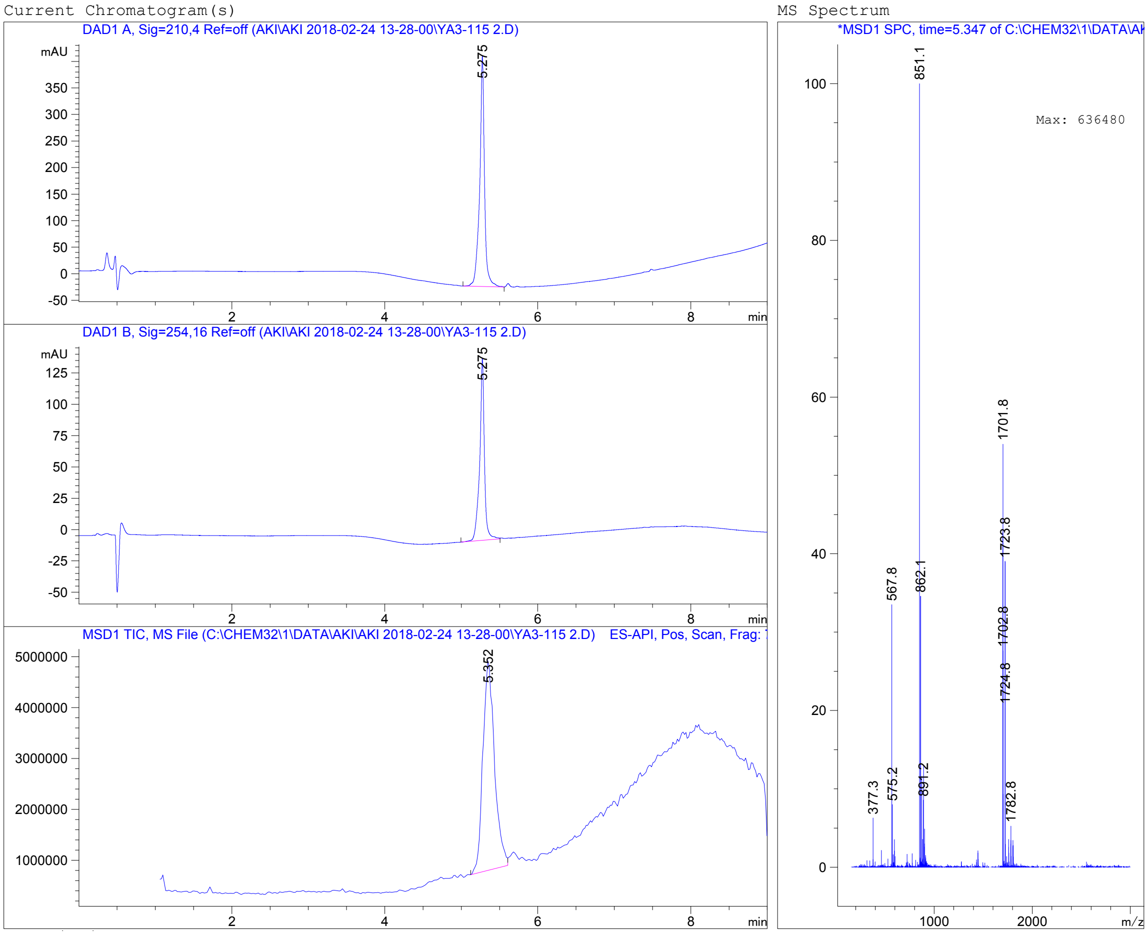

Supplementary Figure 37 HPLC trace and low-resolution ESI-MS spectrum of pure product **S9a**.

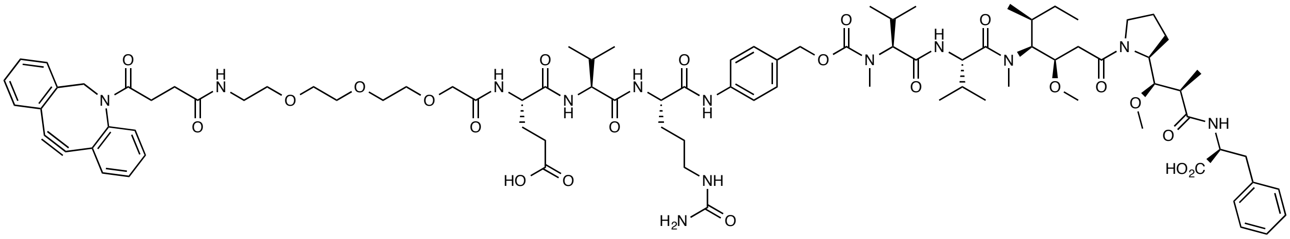

S9b

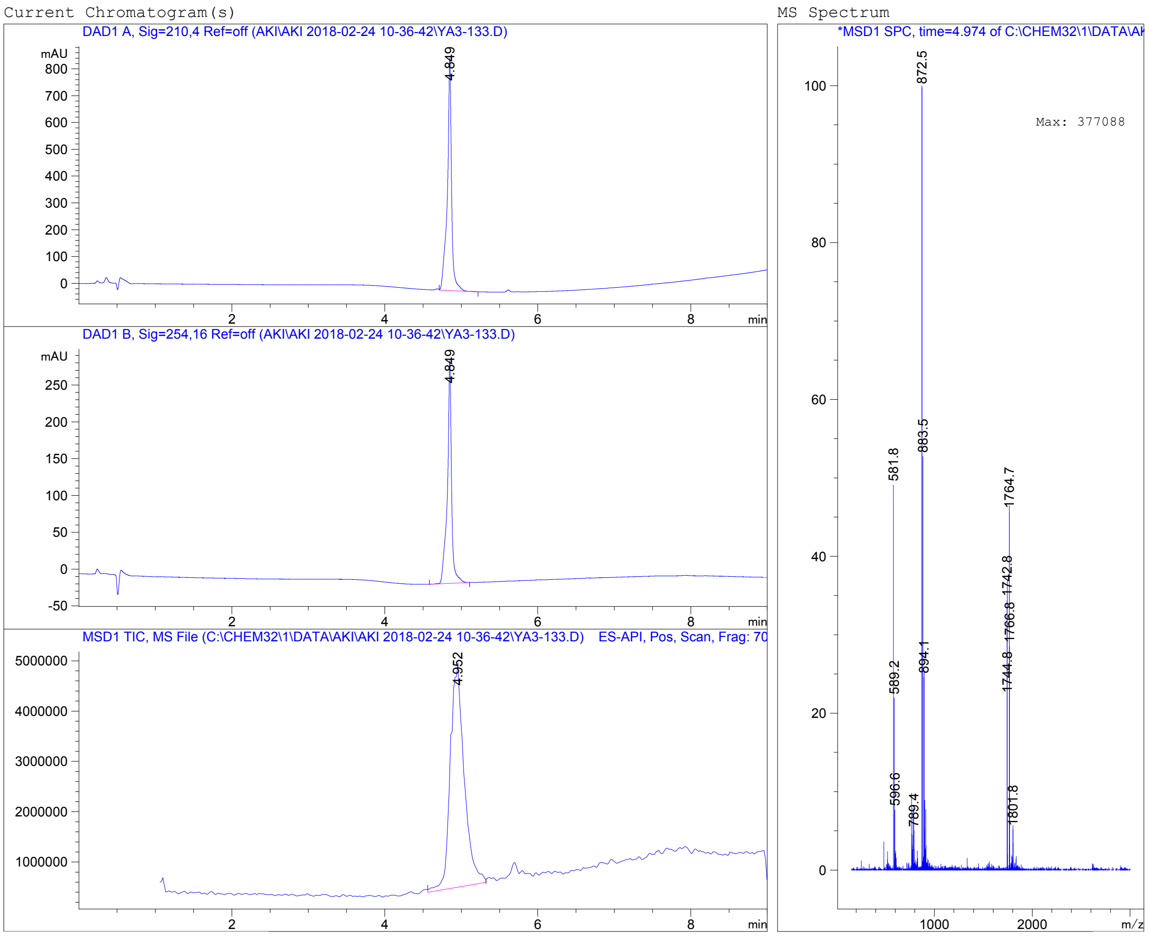

Supplementary Figure 38 HPLC trace and low-resolution ESI-MS spectrum of pure product **S9b**.

Supplementary Tables

Supplementary Table 1 Change of average DARs by incubation with cathepsins. The degree of loss of payload in each ADC was determined by HPLC. All assays were performed in more than twice in technical duplicate.

	Average DAR (mean ± s.e.m., n = 2)			
	5 h		24 h	
	VCit ADC **3a**	EVCit ADC **3c**	VCit ADC **3a**	EVCit ADC **3c**
Cathepsin B	1.78 ± 0.33	0.85 ± 0.25	0.34 ± 0.07	0.08 ± 0.00
Cathepsin L	3.28 ± 0.01	3.64 ± 0.04	2.35 ± 0.04	3.13 ± 0.08
Cathepsin S	1.77 ± 0.24	1.72 ± 0.20	0.55 ± 0.13	0.36 ± 0.07
Cathepsins B+L+S	2.85 ± 0.02	2.80 ± 0.06	1.75 ± 0.01	1.62 ± 0.16

Supplementary Table 2 K_D values of unmodified anti-HER2 mAb and ADCs **3a**–**c** (n = 3). Calculated based on Supplementary Figure 10. Values in parentheses are 95% confidential intervals.

	K_D (nM)	
	KPL-4	MDA-MB-231
N297A anti-HER2 mAb	0.11 (0.092–0.125)	–
VCit ADC **3a**	0.12 (0.114–0.137)	–
SVCit ADC **3b**	0.16 (0.140–0.174)	–
EVCit ADC **3c**	0.14 (0.131–0.161)	–

Supplementary Table 3 EC_{50} values of ADCs in breast cancer cell lines (n = 4). Calculated based on Fig. 3 and Supplementary Fig. 11. Values in parentheses are 95% confidential intervals.

	EC_{50} (nM)				
	KPL-4	BT-474	SKBR-3	JIMT-1	MDA-MB-231
VCit ADC **3a**	0.10 (0.095–0.108)	0.058 (0.051–0.065)	0.27 (0.256–0.289)	0.078 (0.075–0.081)	–
SVCit ADC **3b**	0.12 (0.121–0.127)	0.062 (0.054–0.069)	0.34 (0.321–0.363)	0.102 (0.098–0.105)	–
EVCit ADC **3c**	0.12 (0.116–0.123)	0.063 (0.055–0.072)	0.29 (0.274–0.313)	0.093 (0.089–0.097)	–
Non-cleavable ADC **4**	0.42 (0.399–0.437)	0.247 (0.220–0.278)	0.49 (0.466–0.521)	0.236 (0.219–0.254)	–

Supplementary Table 4 Cell viability at the maximum ADC concentration (n = 4). Calculated based on Fig. 3 and Supplementary Fig. 11. Values in parentheses are 95% confidential intervals.

	Cell viability at the maximum ADC concentration (%)				
	KPL-4	BT-474	SKBR-3	JIMT-1	MDA-MB-231
VCit ADC **3a**	12.2 (11.49–12.91)	25.4 (24.39–26.41)	26.6 (25.60–27.62)	12.2 (11.06–12.63)	–
SVCit ADC **3b**	9.32 (8.823–9.824)	31.9 (30.78–33.02)	25.7 (24.57–26.72)	10.7 (10.17–11.32)	–
EVCit ADC **3c**	11.0 (10.58–11.47)	25.0 (23.64–26.31)	26.3 (25.20–27.30)	11.9 (11.43–12.88)	–
Non-cleavable ADC **4**	15.6 (14.34–16.90)	40.3 (38.71–41.93)	37.5 (36.61–38.38)	27.2 (25.96–28.44)	–

Supplementary Table 5 Summary of in vivo PK parameters (n = 3). Calculated based on Fig. 4a,b and Supplementary Fig. 12. Values in parentheses are 95% confidential intervals. AUC, area under the curve.

	$t_{1/2\beta\ \text{total mAb}}$ (day)	$t_{1/2\beta\ \text{ADC}}$ (day)	$AUC_{\text{total mAb}}$ (h x μg mL^{-1})	AUC_{ADC} (h x μg mL^{-1})
N297A anti-HER2 mAb	14.9	–	3309.6 (2476.8–4140.0)	–
VCit ADC **3a**	14.5	2.0	3775.2 (3316.8–4233.6)	842.4 (763.2–921.6)
SVCit ADC **3b**	12.7	2.4	4948.8 (4449.6–5445.6)	1538.4 (1317.6–1759.2)
EVCit ADC **3c**	14.0	12.0	3907.2 (3283.2–4531.2)	4804.8 (4183.2–5426.4)

Supplementary Table 6 Statistical significance. The *p* values correspond to the asterisks in each figure panel; $*p < 0.025$; $**p < 0.01$; $***p < 0.005$.

Main Figures	Method	Asterisk	Comparison	*p* value
**Fig. 4b**	Welch's *t* test	*<br>*	**3a** *vs.* **3c**<br>**3b** *vs.* **3c**	$p = 0.0141$<br>$p = 0.0146$
**Fig. 4c**	Mann-Whitney *U* test	**<br>**	**3a** 3 mg kg^{-1} *vs.* **3c** 3 mg kg^{-1}<br>**3a** 3 mg kg^{-1} *vs.* **3c** 1 mg kg^{-1}	$p = 0.0079$<br>$p = 0.0079$
**Fig. 4d**	Mann-Whitney *U* test	**	**3a** 3 mg kg^{-1} *vs.* **3c** 3 mg kg^{-1}	$p = 0.0079$
**Fig. 4e**	Log-rank (Mantel-Cox)	*<br>*	**3a** 3 mg kg^{-1} *vs.* **3c** 3 mg kg^{-1}<br>**3a** 3 mg kg^{-1} *vs.* **3c** 1 mg kg^{-1}	$p = 0.0133$<br>$p = 0.0133$
**Fig. 4f**	Log-rank (Mantel-Cox)	***	**3a** 3 mg kg^{-1} *vs.* **3c** 3 mg kg^{-1}	$p = 0.0027$

Supplementary Methods

General information

Unless otherwise noted, all materials for chemical synthesis were purchased from commercial suppliers (Acros Organics, AnaSpec, Broadpharm, Chem-Impex International, Fisher Scientific, Levena Biopharma, Sigma Aldrich, and TCI America) and used as received. All anhydrous solvents were purchased and stored over activated molecular sieves under argon atmosphere.

Analytical thin-layer chromatography (TLC) was performed using silica gel plates (Merck Kieselgel $60F_{254}$, 0.25 mm for TLC) and visualization was conducted with ultraviolet light (254 nm) or by ninhydrin staining. Nuclear magnetic resonance (NMR) spectra were recorded on a Bruker DPX spectrometer (^{1}H: 300 MHz, ^{13}C: 75 MHz) using methanol-d_4 (CD_3OD), dimethyl sulfoxide-d_6 (DMSO-d_6), or deuterium oxide (D_2O) as deuterated solvent. Chemical shifts (δ) in ^{1}H and ^{13}C NMR spectra were reported in parts per million (ppm) relative to CD_3OD (^{1}H: δ = 3.33 ppm, ^{13}C: δ = 49.0 ppm) or DMSO-d_6 (^{1}H: δ = 2.50 ppm, ^{13}C: δ = 39.52 ppm). Coupling constants (J) in all NMR spectra are reported in Hertz (Hz).

Analytical reverse-phase high performance liquid chromatography (RP-HPLC) was performed using an Agilent LC-MS system consisting of a 1100 HPLC and a 1946D single quadrupole electrospray ionization (ESI) mass spectrometer equipped with a C18 reverse-phase column (small molecules: 3×50 mm, 2.6 μm; Accucore C18, Thermo Scientific; antibodies: 3×50 mm, 4 μm; MabPac RP column, Thermo Scientific). Standard analysis conditions for organic molecules were as follows: flow rate = 0.5 mL min^{-1}; solvent A = water containing 0.1% formic acid or 0.1% NH_4OH; solvent B = acetonitrile containing 0.1% formic acid or 0.1% NH_4OH. Compounds were analyzed using a linear gradient and monitored with UV detection at 210 and 254 nm. Preparative HPLC was performed using a Breeze HPLC system (Waters) equipped with a C18 reverse-phase column (19×150 mm, 5.0 μm; SunFire Prep C18 OBD, Waters). Standard purification conditions were as follows: flow rate = 10 mL min^{-1}; solvent A = water containing 0.05% trifluoroacetic acid (TFA) or 0.1% NH_4OH; solvent B = acetonitrile containing 0.05% TFA or 0.1% NH_4OH. Compounds were analyzed using a linear gradient and monitored with UV detection at 210 and 254 nm. In all cases, fractions were analyzed off-line using the LC-MS for purity confirmation and those containing a desired product were lyophilized using a Labconco Freezone 4.5 Liter Benchtop Freeze Dry System. High-resolution mass spectra were obtained using an Agilent 6530 Accurate Mass Q-TOF LC/MS.

Preparation of Fmoc-Cit-PAB-O-resin.

Chlorotrityl chloride resin (1 g, 1.6 mmol) was mixed with a solution of Fmoc-citrulline-PABOH[2] (2.4 g, 4.8 mmol) in pyridine (783 µL, 9.6 mmol), tetrahydrofuran (THF, 30 mL), and dimethylformamide (DMF, 3 mL) and agitated overnight at 55 °C. After the solution was cooled, methanol (MeOH) was added and agitated for 30 min at room temperature. The solution was drained and the resin was washed with DMF (5×5 mL) and dichloromethane (DCM, 5×5 mL). The loading rate was determined to be 10% (0.16 mmol g^{-1} resin) by small-scale resin cleavage using 1% trifluoroacetic acid (TFA)/DCM.

Fmoc Solid-Phase Peptide Synthesis (Fmoc SPPS) for acetyl-capped compounds (S1a–f).

To remove a Fmoc-protecting group after each coupling, resin (100–150 mg) was treated with piperidine (5 mL of 20% in DMF) for 20 min and washed with DMF (5×5 mL) and DCM (5×5 mL). Fmoc-protected amino acid (4 equiv.) was pre-activated by being mixed with 1-[bis(dimethylamino)methylene]-1H-1,2,3-triazolo[4,5-b]pyridinium 3-oxid hexafluorophosphate (HATU, 4 equiv.) and *N*,*N*-diisopropylethylamine (DIPEA, 6 equiv.) in DMF for 2-5 min, and the cocktail was used for coupling (conditions: room temperature, 1 h). The completion of the coupling was verified by the Kaiser test. After each coupling step, the coupling cocktail was drained and the resin was washed with DMF (5×5 mL) and DCM (5×5 mL). After completion of peptide elongation, the resin was treated with acetic anhydride (4 equiv.) and DIPEA (6 equiv.) in DMF for 1 h and then washed with DMF (5×5 mL) and DCM (5×5 mL). The acetyl-capped resin containing protected peptides was treated with 1% TFA/DCM at room temperature for 1 h. The solution was concentrated in vacuo and the crude peptides were precipitated with cold diethyl ether (5–6 mL) followed by centrifugation at 1,000 *g* for 5 min (3 times). The peptide pellet was dried in vacuo and used immediately in the next step without purification.

Ac-Val-Cit-PABC-PNP (S2a).

To a solution of crude **S1a** (3.6 mg, approximately 0.008 mmol) in DMF (0.3 mL) were added bis(2,4-dinitrophenyl) carbonate (12.8 mg, 0.042 mmol) and DIPEA (4.4 µL, 0.025 mmol). After being mixed overnight at room temperature, the crude products were purified by preparative RP-HPLC under acidic conditions to afford peptide **S2a** (2.9 mg, total yield: 62%, based on the resin loading rate). Purity was confirmed by LC-MS under acidic conditions. Off-white powder. HRMS (ESI) Calcd. For $C_{27}H_{34}N_6O_9Na$ $[M+Na]^+$: 609.2279. Found: 609.2286. Peptides **S2b–f** were synthesized from **S1b–f** in a similar manner.

Ac-Ser(*t*-Bu)-Val-Cit-PABC-PNP (S2b).

3.9 mg, total yield: 66% (based on the loading rate). Off-white powder. HRMS (ESI) Calcd. For $C_{34}H_{47}N_7O_{11}Na$ $[M+Na]^+$: 752.3226. Found: 752.3240.

Ac-Glu(*t*-Bu)-Val-Cit-PABC-PNP (S2c).

4.8 mg, total yield: 78% (based on the loading rate). Off-white powder. HRMS (ESI) Calcd. For $C_{36}H_{49}N_7O_{12}Na$ $[M+Na]^+$: 794.3331. Found: 794.3351.

Ac-Asp(*t*-Bu)-Val-Cit-PABC-PNP (S2d).

DMAP (2 equiv.) was used instead of DIPEA. 6.1 mg, total yield: 45% (based on the loading rate). Off-white powder. HRMS (ESI) Calcd. For $C_{35}H_{48}N_7O_{12}$ $[M+H]^+$: 758.3355. Found: 758.3355.

Ac-Lys(Boc)-Val-Cit-PABC-PNP (S2e).

11.1 mg, total yield: 67% (based on the loading rate). Off-white powder. HRMS (ESI) Calcd. For $C_{38}H_{55}N_8O_{12}$ $[M+H]^+$: 815.3934. Found: 815.3931.

***t*-Bu-OCH$_2$CO-Gly-Val-Cit-PABC-PNP (S2f)**.

DMAP (2 equiv.) was used instead of DIPEA. 9.5 mg, total yield: 61% (based on the loading rate). Off-white powder. HRMS (ESI) Calcd. For $C_{33}H_{45}N_7O_{11}Na$ $[M+Na]^+$: 738.3069. Found: 738.3068.

Synthesis of Sarcosine-pyrene (Sar-pyrene, S3).

To a solution of Fmoc-sarcosine-OH (14.0 mg, 0.045 mmol) in DMF (0.5 mL) were added HATU (25.7 mg, 0.0675 mmol), DIPEA (12 µL, 0.0675 mmol), and 1-pyrenemethylamine (13.3 mg, 0.0495 mmol). After being mixed at room temperature for 2 h, the solution was concentrated in vacuo and the crude products were dissolved in DMF (0.6 mL) and diethylamine (0.6 mL). After 1 h, the crude mixture was concentrated and purified by preparative RP-HPLC under acidic conditions to afford sarcosine-pyrene **S3** (6.7 mg, 49% for the 2 steps). White powder. ^{1}H NMR (300 MHz, DMSO-d$_6$) δ 8.82 (t, *J* = 5.8 Hz, 1H), 8.40 (d, *J* = 9.3 Hz, 1H), 8.35–8.23 (m, 4H), 8.17 (s, 2H), 8.14–8.02 (m, 2H), 5.08 (d, *J* = 5.6 Hz, 2H), 3.49 (s, 2H), 2.88 (s, 1H), 2.43 (s, 3H); ^{13}C NMR (75 MHz, DMSO-d$_6$) δ 167.9, 132.4, 130.8, 130.3, 130.2, 128.1, 127.6, 127.4, 127.1, 126.8, 126.3, 125.3, 125.2, 124.7, 124.0, 123.9, 123.1, 51.6, 39.4, 34.3; HRMS (ESI) Calcd. For $C_{20}H_{19}N_2O$ $[M+H]^+$: 303.1492. Found: 303.1492.

Ac-Val-Cit-PABC-Sar-pyrene (1a).

In a microtube were mixed Ac-Val-Cit-PABC-PNP **S2a** (6.3 mg, 10.7 µmol) in DMF (1 mL), 128.4 µL of 100 mM sarcosine-pyrene **S3** in DMF (12.8 µmol), DIPEA (2.8 µL, 16.1µmol), and DMAP (10 µL, 10w/v% in DMF). After being stirred at room temperature for 2 h, the crude products were purified by preparative RP-HPLC under acidic conditions to afford analytically pure product **1a** (2.7 mg, 34%). White powder. HRMS (ESI) Calcd. For $C_{41}H_{47}N_7O_7Na$ $[M+Na]^+$: 772.3429. Found: 772.3440.

Ac-Ser-Val-Cit-PABC-Sar-pyrene (1b).

In a microtube were mixed Ac-Ser(*t*-Bu)-Val-Cit-PABC-PNP **S2b** (6.6 mg, 9.1 µmol) in DMF (1 mL), 108.7 µL of 100 mM sarcosine-pyrene **S3** in DMF (10.9 µmol), DIPEA (2.4 µL, 13.7 µmol), and DMAP (10 µL, 10w/v% in DMF). After being stirred at room temperature for 2 h, the mixture was dried in vacuo and treated with a solution of TFA, DCM, and triisopropylsilane (45:50:5, 2 mL) at room temperature for 3 h. After the solution was concentrated, the crude products were purified by preparative RP-HPLC under acidic conditions to afford analytically pure product **1b** (2.3 mg, 30% for the 2 steps). White powder. HRMS (ESI) Calcd. For $C_{44}H_{52}N_8O_9Na$ $[M+Na]^+$: 859.3749. Found: 859.3753. Probes **1c–f** were synthesized from **S2c–f** in a similar manner.

Ac-Glu-Val-Cit-PABC-Sar-pyrene (1c).

2.6 mg, 31% for the 2 steps. White powder. HRMS (ESI) Calcd. For $C_{46}H_{54}N_8O_{10}Na$ $[M+Na]^+$: 901.3855. Found: 901.3870.

Ac-Asp-Val-Cit-PABC-Sar-pyrene (1d).

3.1 mg, 47% for the 2 steps. White powder. HRMS (ESI) Calcd. For $C_{45}H_{53}N_8O_{10}$ $[M+H]^+$: 865.3879. Found: 865.3877.

Ac-Lys-Val-Cit-PABC-Sar-pyrene (1e).

3.5 mg, 47% for the 2 steps. White powder. HRMS (ESI) Calcd. For $C_{47}H_{60}N_9O_8$ $[M+H]^+$: 878.4559. Found: 878.4553.

$HOCH_2CO$-Gly-Val-Cit-PABC-Sar-pyrene (1f).

2.7 mg, 47% for the 2 steps. White powder. HRMS (ESI) Calcd. For $C_{43}H_{50}N_8O_9Na$ $[M+Na]^+$: 845.3593. Found: 845.3589.

Boc-Lys(Fmoc)-N_3 (S4).

Boc-Lys(Fmoc)-OH (120.4 mg, 0.257 mmol) in DMF (2 mL) was mixed with *N*-hydroxysuccinimide (NHS, 59.2 mg, 0.514 mmol) and *N*-(3-dimethylaminopropyl)-*N'*-ethylcarbodiimide hydrochloride (EDC·HCl, 98.5 mg, 0.514 mmol) at room temperature. To the solution was added 3-azidopropylamine (32.8 μL, 0.334 mmol) and the resulting mixture was stirred overnight at room temperature. The reaction was quenched with 15% citric acid and extracted with ethyl acetate. The organic layer was washed with brine, dried over Na_2SO_4, and concentrated. The residue was purified by column chromatography using a Biotage Isolera Flash Purification System (0–20% of DCM/MeOH, flow rate: 20 mL min^{-1}, SNAP cartridge KP-Sil 10 g) to afford **S4** (178.6 mg, 97%). White powder. ^{1}H NMR (300 MHz, CD_3OD) δ 7.81 (d, *J* = 7.4 Hz, 2H), 7.66 (d, *J* = 7.4 Hz, 2H), 7.41 (t, *J* = 7.3 Hz, 2H), 7.32 (t, *J* = 7.4 Hz, 2H), 4.37 (d, *J* = 6.8 Hz, 2H), 4.21 (t, *J* = 6.9 Hz, 1H), 4.00–3.86 (m, 1H), 3.40–3.34 (m, 2H), 3.27 (td, *J* = 6.6, 2.7 Hz, 2H), 3.12 (t, *J* = 6.7 Hz, 2H), 1.82–1.68 (m, 3H), 1.66–1.48 (m, 3H), 1.44 (s, 9H), 1.40–1.15 (m, 2H); ^{13}C NMR (75 MHz, CD_3OD) δ 175.4, 159.0, 145.4 (2 carbons), 145.3, 142.6 (2 carbons), 128.8 (2 carbons), 128.1 (2 carbons), 126.1 (2 carbons), 120.9 (2 carbons), 80.6, 67.6, 56.3, 50.0, 48.5, 41.3, 37.7, 32.9, 30.5, 29.7, 28.7 (3 carbons), 24.1; HRMS (ESI) Calcd. For $C_{29}H_{38}N_6O_5Na$ $[M+Na]^+$: 573.2796. Found: 573.2802.

N_3-Lys(Fmoc)-N_3 (S5).

Compound **S4** (79 mg, 0.144 mmol) was dissolved in DCM (500 μL) and then TFA (500 μL) was added to the solution at room temperature. After 1 h, the mixture was concentrated and used for the following reaction without purification. To the residue were added azidopropanoic acid (21.5 mg, 0.187 mmol, Advanced ChemBlocks), NHS (33.1 mg, 0.288 mmol), and EDC·HCl (55.2 mg, 0.288 mmol) in DMF (1 mL). DIPEA (50 μL, 0.288 mmol) was subsequently added to the mixture. After being stirred at room temperature overnight, the reaction mixture was quenched with 15% citric acid and extracted with ethyl acetate. The organic layer was washed with brine, dried over Na_2SO_4, and concentrated. The residue was purified by column chromatography using a Biotage Isolera Flash Purification System (0–20% of DCM/MeOH, flow rate: 20 mL min^{-1}, SNAP cartridge KP-Sil 10 g) to afford **S5** (54.9 mg, 70%). White powder. ^{1}H NMR (300 MHz, CD_3OD) δ 8.18 (d, *J* = 7.3 Hz, 1H), 8.03 (t, *J* = 5.8 Hz, 1H), 7.80 (d, *J* = 7.5 Hz, 2H), 7.64 (d, *J* = 7.4 Hz, 2H), 7.39 (t, *J* = 7.1 Hz, 2H), 7.31 (t, *J* = 7.4 Hz, 2H), 4.35 (d, *J* = 6.8 Hz, 2H), 4.32–4.14 (m, 2H), 3.55 (t, *J* = 6.6 Hz, 2H), 3.37–3.32 (m, 2H), 3.25 (q, *J* = 6.2 Hz, 2H), 3.11 (t, *J* = 6.7 Hz, 2H), 2.49 (t, *J* = 6.4 Hz, 2H), 1.82–1.69 (m, 3H), 1.67–1.43 (m, 3H), 1.43–1.27 (m, 2H); ^{13}C NMR (75 MHz, CD_3OD) δ 174.4, 173.0, 159.0, 145.4 (2 carbons), 142.6 (2 carbons), 128.8 (2 carbons), 128.1 (2 carbons), 126.1 (2 carbons), 120.9 (2 carbons), 67.6, 55.0, 50.0, 48.54, 48.51, 41.4,

37.7, 36.0, 32.7, 30.5, 29.6, 24.1; HRMS (ESI) Calcd. For $C_{27}H_{33}N_9O_4Na$ $[M+Na]^+$: 570.2548. Found: 570.2555.

Branched linker (2).

Compound **S5** (29.2 mg, 0.053 mmol) was dissolved in DMF (0.3 mL) and diethylamine (0.3 mL) was added to the solution at room temperature. After 1 h, the mixture was concentrated and purified by preparative RP-HPLC under acidic conditions (UV: 195 nm) to afford branched linker **2** (3.9 mg, 22%). Colorless oil. ^1H NMR (300 MHz, DMSO-d_6) δ 8.17 (d, *J* = 8.0 Hz, 1H), 8.01 (t, *J* = 5.7 Hz, 1H), 7.73 (s, 2H), 4.20 (td, *J* = 8.4, 5.3 Hz, 1H), 3.54–3.49 (m, 2H), 3.34 (t, *J* = 6.8 Hz, 2H), 3.12 (q, *J* = 6.5 Hz, 2H), 2.84–2.66 (m, 2H), 2.45 (t, *J* = 6.4 Hz, 2H), 1.73–1.57 (m, 3H), 1.57–1.43 (m, 3H), 1.41–1.16 (m, 2H); ^{13}C NMR (75 MHz, D_2O) δ 173.9, 173.8, 48.9, 48.6, 47.1, 39.2, 36.6, 34.7, 30.4, 27.6, 26.3, 22.1; HRMS (ESI) Calcd. For $C_{12}H_{23}N_9O_2Na$ $[M+Na]^+$: 348.1867. Found: 348.1868.

Fmoc Solid-Phase Peptide Synthesis (Fmoc SPPS) for DBCO-capped compounds (S6a) and (S6b).

After peptide elongation and pegylation, the resin was treated with DBCO acid (2 equiv., Broadpharm), HATU (2 equiv.), and DIPEA (3 equiv.) in DMF for 1 h and then washed with DMF (5×5 mL) and DCM (5×5 mL). The resin was treated with 1% TFA/DCM at room temperature for 1 h. The solution was concentrated in vacuo and the crude peptides were precipitated with cold diethyl ether (5–6 mL) followed by centrifugation at 1,000 x*g* for 5 min (3 times). The crude products were purified by preparative RP-HPLC under basic conditions. Purity was confirmed by LC-MS under basic conditions.

S6a: 6.4 mg, total yield: 27% (based on the resin loading rate). White powder. HRMS (ESI) Calcd. For $C_{52}H_{70}N_8O_{12}Na$ $[M+Na]^+$: 1021.5005. Found: 1021.4996.

S6b: 7.3 mg, total yield: 29% (based on the resin loading rate). White powder. HRMS (ESI) Calcd. For $C_{54}H_{72}N_8O_{13}Na$ $[M+Na]^+$: 1063.5111. Found: 1063.5131.

DBCO-peg$_3$-Ser(*t*Bu)-Val-Cit-PABC-PNP (S7a).

To a solution of **S6a** (6.2 mg, 0.0062 mmol) in DMF (500 μL) were added bis(2,4-dinitrophenyl) carbonate (9.4 mg, 0.031 mmol) and DMAP (1.5 mg, 0.0124 mmol). The resulting mixture was stirred at room temperature for 2.5 h under Ar. The reaction was quenched with 1% formic acid in ACN and a few drops of MeOH. The crude products were purified by preparative RP-HPLC to afford **S7a** (2.8 mg, 38%). White powder. HRMS (ESI) Calcd. For $C_{59}H_{73}N_9O_{16}Na$ $[M+Na]^+$: 1186.5067. Found: 1186.5075. Peptide **S7b** was prepared from **S6b** in a similar manner.

DBCO-peg$_3$-Glu(*t*Bu)-Val-Cit-PABC-PNP (S7b).

880 µg, 84%. White powder. HRMS (ESI) Calcd. For $C_{61}H_{76}N_9O_{17}$ $[M+H]^+$: 1206.5354. Found: 1206.5388.

DBCO-peg$_3$-Ser(*t*Bu)-Val-Cit-PABC-MMAF (S8a).

A solution of **S7a** (2.8 mg, 0.00238 mmol) in DMF (238 µL) was mixed with monomethyl auristatin F TFA salt (3.0 mg, 0.00357 mmol, Levena Biopharma), 4.76 µL of 1M HOAt in DMF (0.00476 mmol), and DIPEA (2 µL, 0.012 mmol). The resulting mixture was stirred overnight at 37 °C. The crude products were directly purified by preparative RP-HPLC under basic conditions to afford peptide **S8a** (1.1 mg, 26%). Purity was confirmed by LC-MS under basic conditions. White powder. HRMS (ESI) Calcd. For $C_{92}H_{133}N_{13}O_{21}Na$ $[M+Na]^+$: 1778.9631. Found: 1778.9637. Peptide **S8b** was prepared from **S7b** in a similar manner.

DBCO-peg$_3$-Glu(*t*Bu)-Val-Cit-PABC-MMAF (S8b).

1.4 mg, 33%. Purity was confirmed by LC-MS under basic conditions. White powder. HRMS (ESI) Calcd. For $C_{94}H_{135}N_{13}O_{22}Na$ $[M+Na]^+$: 1820.9737. Found: 1820.9706.

DBCO-peg$_3$-Ser-Val-Cit-PABC-MMAF (S9a).

TFA (60 µL) was added to a DCM solution (240 µL) of compound **S8a** (1.1 mg, 0.6 µmol) at 0 °C. After being stirred at 0 °C for 4 h, the reaction mixture was quenched with saturated NH_4OH (300 µL). The resulting mixture was directly purified by preparative RP-HPLC under basic conditions to afford peptide **S9a** (550 µg, 53%). Purity was confirmed by LC-MS under basic conditions. White powder. HRMS (ESI) Calcd. For $C_{88}H_{125}N_{13}O_{21}Na$ $[M+Na]^+$: 1722.9005. Found: 1722.9007. Peptide **S9b** was prepared from **S8b** in a similar manner.

DBCO-peg$_3$-Glu-Val-Cit-PABC-MMAF (S9b).

650 µg, 47%. Purity was confirmed by LC-MS under basic conditions. White powder. HRMS (ESI) Calcd. For $C_{90}H_{127}N_{13}O_{22}Na$ $[M+Na]^+$: 1764.9111. Found: 1764.9072.

Expression and purification of human monoclonal antibodies.

All human monoclonal antibodies were produced according to the procedure reported previously[3,4]. Free style HEK-293 human embryonic kidney cells (Invitrogen) were transfected with a mammalian expression vector encoding for the human IgG1 kappa light chain and full length heavy chain sequences

(based on variable sequences of trastuzumab). A mutation of N297A was incorporated into the heavy chain constant region to produce aglycosylated mAbs. The transfected HEK-293 cells were cultured in a humidified cell culture incubator at 37 °C with 8% CO_2 and shaking at 150 rpm for 7 days before harvesting the culture medium. The antibody secreted into the culture medium was purified using Protein A resin (GE Healthcare). A non-targeting N297A IgG1 (isotype control) was prepared in the same manner.

Supplementary References

1. Dorywalska, M. *et al.* Molecular Basis of Valine-Citrulline-PABC Linker Instability in Site-Specific ADCs and Its Mitigation by Linker Design. *Mol. Cancer Ther.* **15,** 958–970 (2016).
2. Cheng, W. *et al.* Polyconjugates for delivery of RNAi triggers to tumor cells in vivo. *US Patent* US9487556B2 (2016)
3. Shi, Y. *et al.* Engagement of immune effector cells by trastuzumab induces HER2/ERBB2 downregulation in cancer cells through STAT1 activation. *Breast Cancer Res.* **16,** R33 (2014).
4. Anami, Y. *et al.* Enzymatic conjugation using branched linkers for constructing homogeneous antibody-drug conjugates with high potency. *Org. Biomol. Chem.* **15,** 5635–5642 (2017).
